# A biodegradable covalent organic framework for synergistic tumor therapy†

**DOI:** 10.1039/d2sc05732h

**Published:** 2023-01-04

**Authors:** Wen-Yan Li, Jing-Jing Wan, Jing-Lan Kan, Bo Wang, Tian Song, Qun Guan, Le-Le Zhou, Yan-An Li, Yu-Bin Dong

**Affiliations:** a College of Chemistry, Chemical Engineering and Materials Science, Collaborative Innovation Center of Functionalized Probes for Chemical Imaging in Universities of Shandong, Key Laboratory of Molecular and Nano Probes, Ministry of Education, Shandong Normal University Jinan 250014 P. R. China yubindong@sdnu.edu.cn

## Abstract

Stimulus-responsive biodegradable nanocarriers with tumor-selective targeted drug delivery are critical for cancer therapy. Herein, we report for the first time a redox-responsive disulfide-linked porphyrin covalent organic framework (COF) that can be nanocrystallized by glutathione (GSH)-triggered biodegradation. After loading 5-fluorouracil (5-Fu), the generated nanoscale COF-based multifunctional nanoagent can be further effectively dissociated by endogenous GSH in tumor cells, releasing 5-Fu efficiently to achieve selective chemotherapy on tumor cells. Together with the GSH depletion-enhanced photodynamic therapy (PDT), an ideal synergistic tumor therapy for MCF-7 breast cancer *via* ferroptosis is achieved. In this research, the therapeutic efficacy was significantly improved in terms of enhanced combined anti-tumor efficiency and reduced side effects by responding to significant abnormalities such as high concentrations of GSH in the tumor microenvironment (TME).

## Abstract 2

Stimulus-responsive biodegradable nanocarriers with tumor-selective targeted drug delivery are critical for cancer therapy.

## Graphical abstract

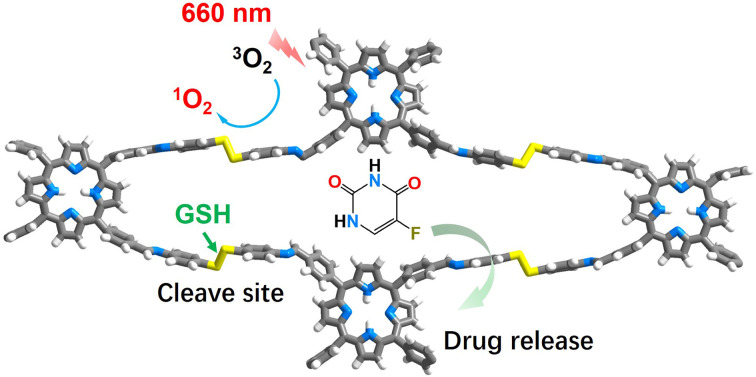

## Introduction

Since the pioneering work of Yaghi *et al.* in 2005,^1,2^ covalent organic frameworks (COFs), as an emerging class of crystalline polymeric materials with porous structures, have shown great potential in the biomedical field, especially in tumor nanotherapeutics.^3–6^ Due to their high stability, inherent porosity, and easy functionalization, COFs have become a promising class of candidates for powerful nanosystems to realize multifunctional integration for synergistic tumor therapy. Furthermore, their metal-free nature prevents any potential biological toxicity caused by metal species.^7–9^

On the other hand, the polymeric crystalline COFs derived from covalent bonding interactions are usually resistant to physical aging and biological attacks. The enrichment of these exogenous nanoscale COF (NCOF) particles in the body may cause serious health risks. Additionally, the “long-lived” artificial organic polymers can cause severe pollution to the environment resulting from their poor recycling and improper disposal.

As we know, one of the most common NCOF-based tumor treatments is drug delivery.^10–24^ The strong host–guest interactions between COF carriers and loaded organic drug molecules might sometimes cause difficulty for payload release under physiological conditions within a limited period, which has often been observed in COF-based drug delivery systems.^10–24^ Large aggregates are formed in a synchronous pathway after exfoliation around the drug-loaded COF crystals, which might limit drug release.^25^ However, all these time-limited applications require the elimination of the artificial NCOFs after use to restore the surrounding living medium to that of a normal organism. From this viewpoint, biodegradable COFs, especially endogenous cellular stimuli-responsive biodegradable ones, are highly desirable.

Disulfides, as an important class of compounds, are widely present in natural products,^26,27^ pharmaceuticals,^28,29^ and functional materials,^30,31^ and they also play a central role in cellular redox homeostasis. For example, the redox balance between glutathione disulfides (GSSG) and glutathione (GSH) is essential for cell growth and function.^32^ Not only that, disulfide is a typical degradable functional group in organic polymers, and its dynamic nature accounts for unique properties of adaptability, degradability, stress resistance, and self-healing in response to extrinsic chemical or physical stimulations.^33^ For example, it can be easily cleaved into two thiol groups under reductive conditions.^34^

Glutathione (GSH), as the most abundant nonprotein molecule in tumor cells, is closely associated with cancer progression and chemoresistance because of its sensitive response to intracellular oxidative stress.^35–37^ It has been reported that the GSH level in tumor cells is 100–1000 fold higher than that in normal cells.^38,39^ Thus, a high level of GSH has been recognized as a specific tumor endogenous index to distinguish between normal and tumor cells.

Inspired by the reported results, we envision that biodegradable NCOF-carriers could be achieved by the synthesis of disulfide-involved COFs *via* ingenious reticular design.^40^ By incorporating additional functions such as phototherapy into NCOFs, the generated multifunctional integrated biodegradable NCOF-nanoagent would meet the multifaceted requirements of selective tumor nanotherapeutics. First, the disulfide-connected NCOFs are stable under physiological conditions and can protect loaded molecular drugs from extracellular degradation; furthermore, they show improved endocytosis-based cellular uptake; second, disulfide is a redox-response functional group that can be readily cleaved upon reduction; third, GSH, as an endogenous tumor stimulator, can trigger disulfide cleavage by reduction to lead to COF-framework disassembly upon tumor cell uptake, consequently enabling a smooth drug release; fourth, GSH depletion will enhance intracellular reactive oxygen species (ROS) levels and guarantee the antitumor efficacy of photodynamic therapy (PDT).^35–37,41–43^ Due to the significant difference in GSH levels between normal and tumor cells, the added therapeutic benefits on tumors with overexpressed GSH could be achieved *via* selectively improving the release of payloads in the tumor microenvironment.

**Scheme 1:**
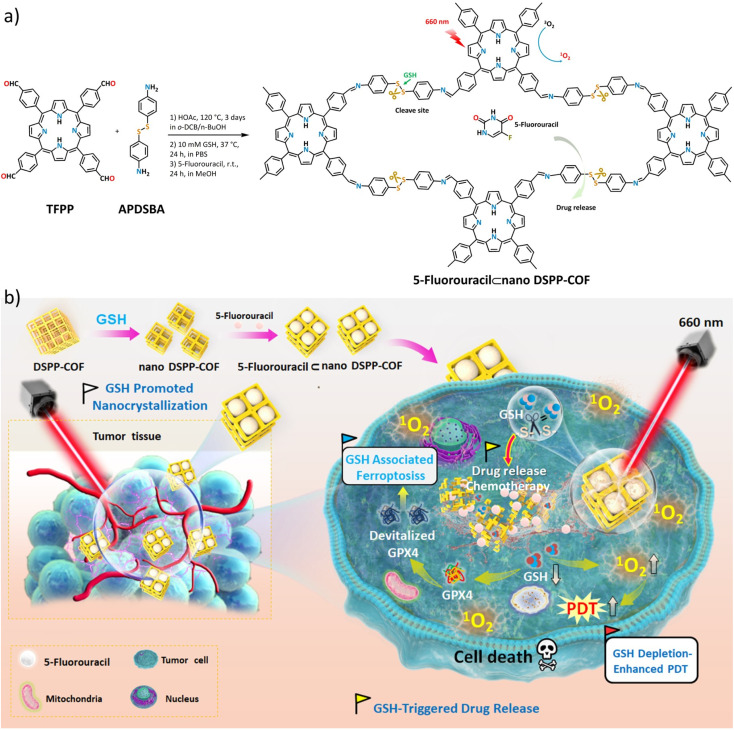
(a) Synthesis of DSPP-COF, nano DSPP-COF, and 5-Fu⊂nano DSPP-COF. (b) Synthesis and treatment application of 5-Fu⊂nano DSPP-COF, including GSH-promoted DSPP-COF nanocrystallization, 5-Fu loading, and the obtained 5-Fu⊂nano DSPP-COF for combination antitumor treatment by endogenous GSH-triggered drug release and GSH depletion-enhanced PDT *via* the ferroptosis pathway.

In this contribution, we report, the first of its kind, a disulfide-connected and metal-free porphyrin-NCOF, and its 5-fluorouracil (5-Fu)-loaded nanoagent (Scheme 1a). The obtained 5-Fu⊂nano DSPP-COF can be effectively degraded by the endogenous GSH of tumor cells along with a highly efficient 5-Fu release under physiological conditions. Together with the GSH depletion-promoted PDT, an ideal tumor synergistic therapy for MCF-7 breast cancer *via* the ferroptosis mechanism was achieved (Scheme 1b), which was fully evidenced by *in vitro* assays and *in vivo* experiments.

## Results and discussion

**Fig. 1:**
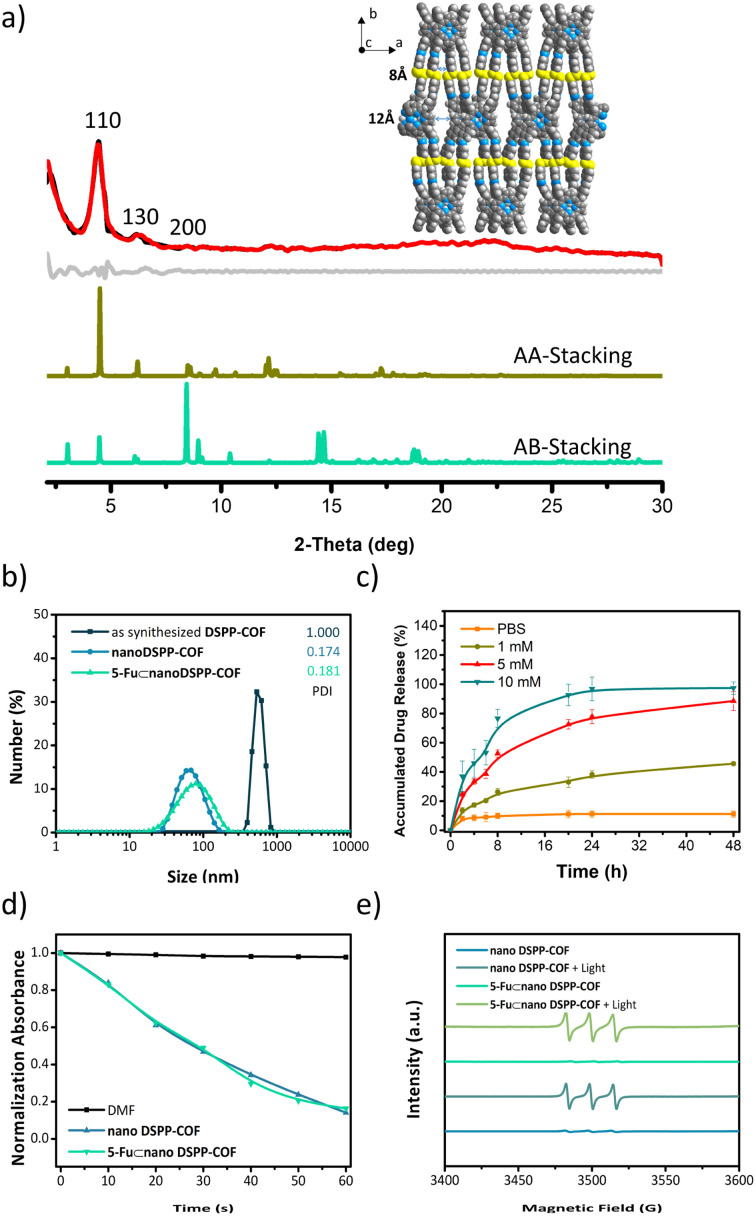
(a) Indexed experimental (red), Pawley-refined (black), and simulated (dark yellow: AA-Stacking; green: AB-stacking) PXRD patterns of DSPP-COF. The difference plot is presented in light gray. Inset shows the crystal AA-stacking structure of DSPP-COF (C, dark gray; N, blue; S, yellow; H, light gray). (b) DLS plots and PDI of as-synthesized DSPP-COF, nano DSPP-COF, and 5-Fu⊂nano DSPP-COF. (c) 5-Fu release behaviour triggered by GSH with different concentrations. Data are presented as the mean ± SD (*n* = 3). (d) Comparison of absorbance decay rates of DPBF at 412 nm induced by as-synthesized nano DSPP-COF (50 μg mL^−1^), 5-Fu⊂nano DSPP-COF (50 μg mL^−1^), and DMF (2 mL) under 660 nm LED irradiation (50 mW cm^−2^). (e) EPR spectrum of ^1^O_2_ trapped by TEMP with/without irradiation (660 nm, 50 mW cm^−2^, 1 min).

As shown in Scheme 1a, the disulfide-COF, termed DSPP-COF, was synthesized by imine condensation of 5,10,15,20-tetrakis(4-formylphenyl)porphyrin (TFPP) and 4-(2-(4-aminophenyl)disulfanyl)benzenamine (APDSBA) under solvothermal conditions (1,2-dichlorobenzene/*n*-BuOH, HOAc, 120 °C, 3 days) in 79% yield (ESI†). The formation of the imine-linked disulfide-COF was well-verified by FT-IR (Fig. S1a, ESI†) and ^13^C CP-MAS solid-state NMR spectroscopy (Fig. S1b, ESI†). Thermogravimetric analysis (TGA) indicated that it is thermally stable up to ∼500 °C (Fig. S1c, ESI†). DSPP-COF possesses good crystallinity, as revealed by the powder X-ray diffraction (PXRD) pattern. As shown in Fig. 1a, DSPP-COF exhibited a series of intense peaks at 4.4°, 6.2°, and 8.6° that correspond to the (110), (130), and (200) planes, respectively. Structural modeling was then conducted with the software Materials Studio.^44^ The most probable structure of DSPP-COF was simulated (Fig. 1a), analogous to that of DSPP-COF, as 2D layered sheets with an eclipsed (AA) stacking mode using the hexagonal space group *P*1 with the optimized parameters *a* = 20.84 Å, *b* = 58.51 Å and *c* = 3.41 Å (residuals *R*_wp_ = 4.31% and *R*_p_ = 2.64%, Table S1, ESI†).

The porosity of DSPP-COF was examined using gas adsorption–desorption measurement, and its sorption profile can be described as a type IV isotherm, which is characteristic of mesoporous materials. N_2_ adsorption at 77 K revealed absorption of 750 cm^3^ g^−1^ for DSPP-COF (Fig. S1d, ESI†), and its surface area calculated on the basis of the BET model was determined to be 477 m^2^ g^−1^. The pore size distribution curves, plotted *via* nonlocal density functional theory (NLDFT) analysis (Fig. S1e, ESI†), showed that the pore width distribution of DSPP-COF is centered at ∼0.8 and 1.2 nm, which is well consistent with its crystal structure.

Scanning electron microscopy (SEM) (Fig. S2, ESI†) showed that DSPP-COF was a microcrystalline material, which was further verified by dynamic light scattering (DLS) analysis (particle size distribution centered at *ca.* 533 nm, polydispersity index (PDI) of DSPP-COF: 1.000, Fig. 1b, and S1f, ESI†). As we know, microscale particles with low aqueous dispersibility are not conducive to cell uptake and cannot be directly applied to tumor treatment.^3–6^ Of note, DSPP-COF herein is a dynamic disulfide-connected framework that can be easily cleaved by a reductant, which was well demonstrated by the model reaction of 4,4′-bis-benzylidene-amino-diphenyl-disulfide and GSH in PBS (37 °C, 12 h, Fig. S3, ESI†). The reduction product of 4-(benzylideneamino)benzenethiol was obtained in 93% yield under the given conditions, indicating that GSH is an effective reducing agent to break the disulfide bond under physiological conditions. On the basis of this, we performed the GSH-triggered reduction of the as-synthesized DSPP-COF. The detailed study indicated that this biodegradation behaviour of disulfide-COF was GSH concentration- and time-dependent, that is, a higher degree of degradation was observed with an increase in GSH concentration and reaction time (Fig. S4, ESI†). According to the obtained results, together with the actual GSH amount in tumor cells, the optimized DSPP-COF degradation conditions were set as: 10 mM GSH, 37 °C, and 24 h, in PBS. Just as expected, *ca.* 59 nm nanoscale DSPP-COF particles were obtained under the optimized conditions, and the PDI of the nanoscale DSPP-COF was 0.174 (Fig. 1b, and S5a, ESI†), which is the perfect size for biomedical applications. This is different from the reported COF nanocrystallization methods;^3^ the redox chemistry derived degradation described herein is a new approach to access NCOFs.

After degradation, nano DSPP-COF still possesses its original structure, high crystallinity, and porosity (Fig. S5b–e, ESI†). The sulfhydryl group content on the surface of nano DSPP-COF was measured by Ellman's method to be 0.138 μmol mg^−1^, so the percentage of disulfide bond breakage in DSPP-COF was 8.12% after 24 h of reaction in 10 mM GSH solution (Fig. S6, ESI†).

As shown in Fig. 1a, the suitable pore size with the enriched heteroatoms should allow the porous DSPP-COF to be a perfect carrier to load 5-Fu molecules (5.5 Å) through host–guest interactions. Indeed, by immersing nano DSPP-COF in a MeOH solution of 5-Fu at room temperature for 24 h, the host–guest supramolecular system of 5-Fu⊂nano DSPP-COF (*ca.* 77 nm in size, PDI at 0.181, Fig. 1b and S7a, ESI†) was readily obtained, which was evidenced by the UV-vis, IR, and porosity studies (Fig. S7b–g, ESI†). The loaded content of 5-Fu in 5-Fu⊂nano DSPP-COF was determined to be ∼0.86 μmol mg^−1^ by the standard curve method, where the standard curves were derived from UV absorption methods, respectively (Fig. S8, ESI†). Of note, the particle size distribution of 5-Fu⊂nano DSPP-COF was basically unchanged in PBS at different pH values (7.4, 6.5, and 5.0) even after 12 days (Fig. S9, ESI†), indicating its excellent long-term stability under physiological conditions. In addition, its negatively charged surface (Fig. S7g, ESI†), together with the suitable nanoscale size, would significantly facilitate its accumulation at tumor sites through the enhanced permeability and retention effect (EPR).^45^ In addition, the GSH-triggered biodegradable COF provided a unique platform for drug delivery. In principle, the susceptibility of DSPP-COF to GSH should be influenced by its content, with a higher GSH concentration causing more GSH-triggered 5-Fu release. Fig. 1c and S10 (ESI†) show that up to ∼96.9% of the encapsulated 5-Fu was released when 5-Fu⊂nano DSPP-COF was placed in a 10 mM GSH buffered solution for 24 h, while only ∼11.3% 5-Fu delivery was observed without GSH. Thus, the GSH-mediated COF disassembly is the key to this drug release; furthermore, this should selectively occur in tumor cells with high GSH concentrations, which potentially confers a tumor cell targeting property to DSPP-COF.

Besides drug release, the porphyrin-containing COF still possesses ^1^O_2_ generation ability. As shown in Fig. 1d, in the presence of nano DSPP-COF, the absorbance of DPBF at 412 nm was reduced to *ca.* 16% of the initial value upon 660 nm LED irradiation (50 mW cm^−2^) for 1 min, implying its highly efficient ^1^O_2_ generation. Compared with nano DSPP-COF, 5-Fu⊂nano DSPP-COF exhibited the same ^1^O_2_ generation efficiency, indicating that the loaded 5-Fu did not affect ^1^O_2_ generation ability. In addition, the light-induced ^1^O_2_ generation of nano DSPP-COF and 5-Fu⊂nano DSPP-COF was further verified by their electron paramagnetic resonance (EPR) spin trapping spectra,^46^ wherein the characteristic peaks of the 2,2,6,6-tetra-methylpiperidine (TEMP)-^1^O_2_ adduct were observed; meanwhile, no TEMP-^1^O_2_ signals were detected in the dark under the same conditions (Fig. 1e).

**Fig. 2:**
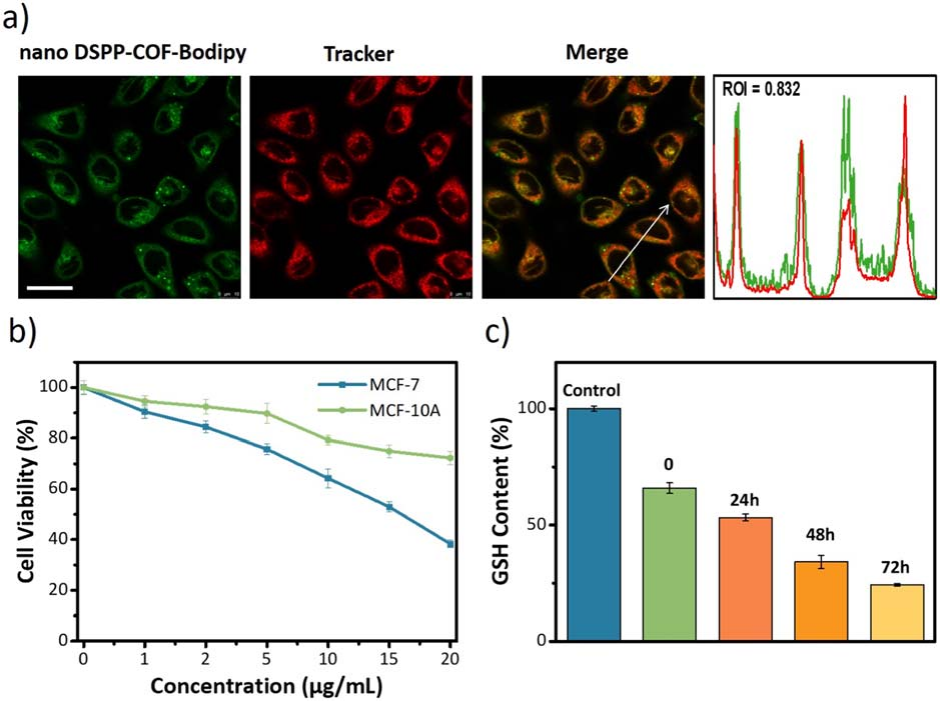
(a) Distribution of nano DSPP-COF-Bodipy in the mitochondria of MCF-7 cells using a mitochondrial tracker. Scale bar: 25 μm. (b) Cell viabilities of MCF-7 and MCF-10A cells treated with 5-Fu⊂nano DSPP-COF for 4 h and cultured for an additional 72 h. (c) The GSH content in MCF-7 cells. MCF-7 cells were co-incubated with nano DSPP-COF for 4 h (noted as 0), and then nano DSPP-COF was removed and the cells were incubated for 24, 48, and 72 h.

**Fig. 3:**
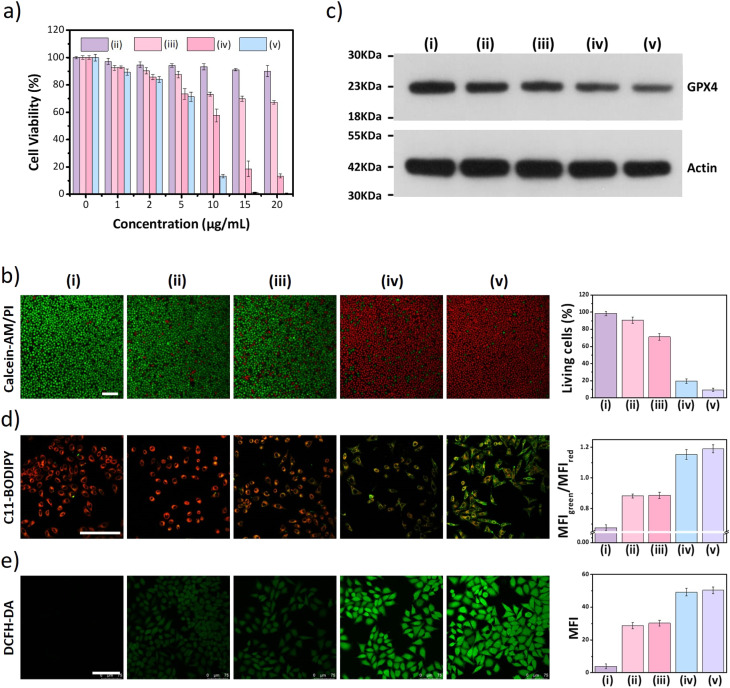
(a) Cell viabilities of MCF-7. (b) Calcein-AM/PI double staining. (c) Expression of GPX4 in MCF-7 cells. (d) LCSM images of MCF-7 cells under different treatment conditions for the detection of intracellular lipid peroxidation using C_11_-Bodipy. (e) Detection of intracellular ^1^O_2_ using DCFH-DA. The scale bar in figures (b), (d), and (e) is 100 μm. (i–v) In the figures represent (i) control, (ii) nano DSPP-COF, (iii) 5-Fu⊂nano DSPP-COF, (iv) nano DSPP-COF + light, and (v) 5-Fu⊂nano DSPP-COF + light, respectively.

Next, the distribution of nano DSPP-COF in MCF-7 cells was examined. By an aldimine condensation reaction between the aldehyde groups in Bodipy-CHO and the free amino groups on nano DSPP-COF (Fig. S11, ESI†), the Bodipy-labelled and structure-preserved nano DSPP-COF for subcellular localization analysis was generated. The obtained results showed a Pearson co-localization coefficient of 0.832 for mitochondria (Fig. 2a), indicating that the nano COF particles were enriched in mitochondria after cell uptake. Internalization mechanism exploration experiments showed that low temperature (4 °C), dichloroacetate (DCA, inhibiting aerobic glycolysis through inhibiting pyruvate dehydrogenase kinase), and methyl-β-cyclodextrin (MβCD, caveolin-dependent endocytosis inhibitor) significantly inhibited the uptake of nano DSPP-COF by MCF-7. This suggests that the pathway of nano DSPP-COF uptake by MCF-7 is through caveolin-dependent endocytosis (Fig. S12, ESI†). Compared with normal MCF-10A cells, 5-Fu⊂nano DSPP-COF displayed stronger dark toxicity toward MCF-7 tumor cells (Fig. 2b), demonstrating that the DSPP-COF carrier was more degradable in MCF-7 cells with higher endogenous GSH levels leading to a greater 5-Fu release. Of course, this intracellular COF degradation was accompanied by GSH depletion, and *ca.* 76% GSH in MCF-7 cells was consumed after 72 h (Fig. 2c). This differential degradation behaviour of the disulfide-COF carrier driven by endogenous GSH between tumor and normal cells would dramatically improve the selectivity and biosafety of nano DSPP-COF in practical tumor treatment. As shown in Fig. 3a, CCK-8 cell viability assay showed that MCF-7 cell viability in the presence of nano DSPP-COF was *ca.* 90%, even with a high concentration up to 20 μg mL^−1^, indicating its negligible dark toxicity and good biocompatibility. In contrast, 5-Fu⊂nano DSPP-COF (20 μg mL^−1^) reduced MCF-7 cell viability to *ca.* 68% in the dark, suggesting that the released 5-Fu *via* GSH-triggered COF degradation could effectively kill tumor cells *via* chemotherapy. Next, the toxicity of 5-Fu at the same concentration as in 5-Fu⊂nano DSPP-COF was examined. 5-Fu exhibited almost the same cytotoxicity as 5-Fu⊂nano DSPP-COF under dark conditions (Fig. S13, ESI†). It was further found that 5-Fu in 5-Fu⊂nano DSPP-COF could be efficiently released in the presence of high concentrations of GSH. A significant decrease in cell viability (*ca.* 13%) was observed with nano DSPP-COF (20 μg mL^−1^) under light irradiation (red LED, 50 mW cm^−2^, 8 min), implying that nano DSPP-COF possessed high phototoxicity toward MCF-7 cells. This powerful antitumor efficacy exhibited by nano DSPP-COF clearly resulted from the enhanced photodynamic therapy (PDT) *via* intracellular GSH-depletion^37,41^ caused by the DSPP-COF degradation. After replenishing with GSH-OEt (a GSH precursor), the phototoxicities of both nano DSPP-COF and 5-Fu⊂nano DSPP-COF towards MCF-7 cells were obviously weakened under the same conditions, fully evidencing this GSH depletion-enhanced PDT (Fig. S14, ESI†). Logically, the best antitumor effect was obtained in the 5-Fu⊂nano DSPP-COF (20 μg mL^−1^) treated group under light irradiation, suggesting a combined effect of 5-Fu-based chemotherapy and porphyrin-derived PDT. This observation was further confirmed by the live-dead cell staining (AM/PI double staining) results (Fig. 3b). On the other hand, GSH is closely associated with cell death, especially in the ferroptosis pathway.^47–49^ As we know, the most important feature of ferroptosis is lipid peroxidation, in which GPX4 plays a crucial role in defective lipid peroxide repair. The intracellular GSH depletion, however, inhibits GPX4 expression, which can effectively reduce the repair of lipid peroxidation.^50^ The detailed cell death mechanism revealed that nano DSPP-COF and 5-Fu⊂nano DSPP-COF caused cell death under given conditions was significantly alleviated by the ferroptosis inhibitors ferrostatin-1 (Fer-1) and liproxstatin-1 (Lip-1), whereas the necroptosis and autophagy inhibitors of necrostatin-1 and 3-methyladenine had no detectable effects on the cell death under the same conditions (Fig. S15, ESI†). These findings imply that nano DSPP-COF and 5-Fu⊂nano DSPP-COF exposure led to reduced cell viability through the ferroptosis pathway. Western blot analysis of GPX4 (Fig. 3c) and assays of GPX4 activity (Fig. S16, ESI†) indicated that GSH depletion inhibited GPX4 expression and that 5-Fu also inhibited GPX4 expression to some extent, thereby attenuating GPX4 rescue of ferroptosis.

The degree of lipid peroxidation in cells was quantified by the C_11_-BODIPY fluorescent probe. As shown in Fig. 3d, the red fluorescence of C_11_-BODIPY was converted to green after the lipids were oxidized. The addition of Fer-1 significantly reduced the degree of lipid peroxidation (Fig. S17, ESI†). Then, ROS were detected in the cells using the DCFH-DA probe by LCSM imaging, and an increase in ROS contributing to an upregulation of the degree of ferroptosis was clearly observed (Fig. 3e). However, Fer-1 could effectively decrease the ROS level in MCF-7 cells (Fig. S18, ESI†), which is responsible for the reduced degree of lipid peroxidation.

In addition, JC-1 staining indicated that the mitochondrial membrane potential was almost completely lost after nano DSPP-COF or 5-Fu⊂nano DSPP-COF treatment in light (Fig. S19, ESI†), as evidenced by the change in JC-1 from J-aggregates (red) to monomers (green).^51,52^ Additionally, a lysosomal permeability change based on acridine orange (AO) staining was observed; the decreased red fluorescence and increased green fluorescence indicated an increase in lysosomal membrane permeabilization under the given conditions (Fig. S20, ESI†). The damage to mitochondria and lysosomes by the nanodrug under light conditions was significantly reduced by Fer-1, thus making the cell-killing effect of the nanodrug less effective (Fig. S21, ESI†). Taken together, these results indicated that lipid peroxidation inflicts irreversible damage to some subcellular structures, which in turn impairs their function and allows the tumor cells to eventually die in a ferroptosis manner.

**Fig. 4:**
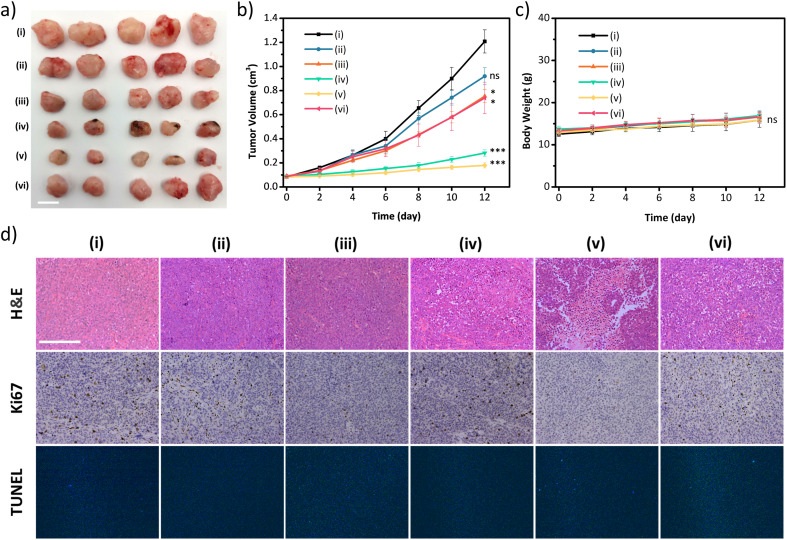
(a) Photographs of tumor tissue after treatment. Scale bar: 1 cm. (b) Tumor volume after treatment. (c) Bodyweight of the mice in various groups during the treatment. (d) Representative images of H&E, Ki67, and TUNEL staining of tumor tissues were obtained at the treatment endpoint, scale bar: 200 μm. (i–vi) In the figure represent (i) control, (ii) nanoDSPP-COF, (iii) 5-Fu⊂nano DSPP-COF, (iv) nanoDSPP-COF + light, (v) 5-Fu⊂nano DSPP-COF + light, and (vi) 5-Fu⊂nano DSPP-COF + light + Fer-1, respectively. All data are presented as the mean ± SD (*n* = 5). **p <0.01; *p <0.05; ns, no significance (*p* >0.05).

The excellent *in vitro* therapeutic efficacy of the biodegradable disulfide-COF-based nanoagent for MCF-7 encouraged us to further evaluate its antitumor ability *in vivo*. Of note, no hemolysis was observed even for the concentration of 5-Fu⊂nano DSPP-COF of 200 μg mL^−1^ (Fig. S22, ESI†), indicating that 5-Fu⊂nano DSPP-COF possessed satisfactory biocompatibility and could be applied in *in vivo* experiments. *In vivo* biodistribution analysis was carried out using 5-Fu⊂nano DSPP-COF-Cy5 (Fig. S23, ESI†), and the biodistribution experiments revealed that the nanoagent was predominantly distributed in the tumor within 24 h of intratumoral injection, as illustrated by *in vivo* fluorescence imaging and *ex vivo* organ imaging (Fig. S24a and b, ESI†). Based on the fluorescence imaging results of intratumoral injection within 24 h, it can be concluded that intratumoral injection can maximize the accumulation of nanodrugs at the tumor site, minimize acute toxicity, and reduce side effects in other organs. With the extension of imaging time, it was found that the fluorescence at the tumor site gradually diminished, indicating that the GSH-triggered biodegradable nanodrug was gradually excreted from the nude mice, which is beneficial for the biosafety of nanodrugs (Fig. S24c, ESI†). The *in vivo* antitumor ability was investigated in an MCF-7 xenograft nude mouse model. Compared to the control group (i), almost no antitumor effect was observed in the nano DSPP-COF treated group (ii) in the dark, indicating that nano DSPP-COF possessed no dark toxicity. In contrast, the tumor volumes of the 5-Fu⊂nano DSPP-COF in the dark (iii) and nano DSPP-COF + light (iv) groups were *ca.* 57% and 22% of that of the control group respectively, indicating their effective antitumor effects under the given conditions. To our delight, the tumor volume in the 5-Fu⊂nano DSPP-COF + light group (v) was only *ca.* 14% of that of the control group, implying that the combination of PDT and chemotherapy provided the best therapeutic effect and significantly inhibited tumor growth (Fig. 4a and b).

In order to further verify that the tumor growth inhibition herein was caused by ferroptosis, intra-tumoral injection of Ferrostatin-1 (Fer-1), a ferroptosis inhibitor, was administered along with 5-Fu⊂nano DSPP-COF (vi) to block lipid peroxidation, indicating that the inhibitory effect on tumors was significantly weakened under the given conditions. This observation fully confirmed that the synergistic antitumor activity exhibited by 5-Fu⊂nano DSPP-COF against breast cancer occurred through the ferroptosis mechanism (Fig. 4a and b). The robust weight gain of the nude mice in the experimental groups was attributed to the use of PDT to avert systemic toxicity through local light exposure at the tumor site (Fig. 4c). This was further validated by the absence of significant material-induced damage and inflammation in the H&E staining of the major organs of the nude mice (Fig. S25, ESI†). H&E staining of tumor tissues showed that group (v) experienced the most severe cell death. Ki67 staining showed that group (v) exhibited the lowest proliferative potential. The tunel staining result of the treated group was almost identical to that of the control group, which indicated that damage of DNA in the nucleus of the tumor cells caused by the material was negligible (Fig. 4d and S26, ESI†).

## Conclusion

In conclusion, we have developed a new type of biocompatible, biodegradable, and fast redox-responsive COF-based multifunctional nanoagent. The resulting drug loaded NCOF was GSH-responsive and could be disintegrated in the tumor microenvironment by high-level GSH, and exhibited selective drug release upon reduction of the disulfide bonds after tumor cell uptake. Together with the GSH depletion-enhanced PDT, a powerful combination therapy of chemotherapy and PDT for tumor treatment *via* ferroptosis was achieved. We believe that tumor microenvironment-responsive NCOFs could provide more possibilities to obtain multifunctional nanoagents that enable a safer way to achieve precise and efficient tumor treatment. We expect that our research can promote the development of reticular framework materials, especially NCOFs, in the biomedical field. Our research represents a major step forward in the use of reticular design to make active nanocarrier scaffolds.

## Data availability

The data that support the findings of this study are presented in the paper and the ESI.†

## Author contributions

All authors contributed extensively to the work presented in this paper. Conceptualization, Y.-B. Dong. The investigation, W.-Y. Li, J.-J. Wan, J.-L. Kan, B. Wang, T. Song, Q. Guan, L.-L. Zhou, and Y.-A. Li. Methodology, W.-Y. Li, Y.-A. Li and Y.-B. Dong. Project administration, Y.-B. Dong. Resources, Y.-B. Dong. Supervision, Y.-B. Dong. Visualization, W.-Y. Li and Y.-B. Dong Writing – original draft, W.-Y. Li. Writing – review & editing, W.-Y. Li and Y.-B. Dong.

## Conflicts of interest

There are no conflicts to declare.

## Supplementary Material

SC-014-D2SC05732H-s001
