# Supplementary material for: A biodegradable covalent organic framework for synergistic tumor therapy
Source: Chem Sci. 2023 Jan 4;14(6):1453–60. doi: 10.1039/d2sc05732h (PMC9906711; doi:10.1039/d2sc05732h)
Supplement: SC-014-D2SC05732H-s001 [file SC-014-D2SC05732H-s001.pdf]

Electronic Supplementary Material (ESI) for Chemical Science.
This journal is © The Royal Society of Chemistry 2023

A biodegradable covalent organic framework for synergistic tumor therapy

Wen-Yan Li, Jing-Jing Wan, Jing-Lan Kan, Bo Wang, Tian Song, Qun Guan, Le-Le Zhou, Yan-An Li and Yu-Bin Dong*

College of Chemistry, Chemical Engineering and Materials Science, Collaborative Innovation Center of Functionalized Probes for Chemical Imaging in Universities of Shandong, Key Laboratory of Molecular and Nano Probes, Ministry of Education, Shandong Normal University, Jinan 250014, P. R. China. Email: yubindong@sdnu.edu.cn

1. Experimental Materials 2
2. Cell Culture and Experimental Animals..2
3. Experimental Instrumentations..3
4. Statistical Analysis 4
5. Synthesis of DSPP-COF 4
6. Model Reaction 18
7. Synthesis of nano DSPP-COF 19
8. Determination of sulfhydryl groups on nano DSPP-COF by Ellman's method 20
9. Drug loading experiments 21
10. Drug Release Experiments 22
11. ESR-Trapping Test..23
12. Photodynamic Property 23
13. Cell Uptake and Subcellular Localization..24
14. Cellular Uptake Mechanism..24
15. Intracellular GSH Measurements 25
16. CCK-8 Cell Viability Assays 25
17. Calcein-AM/PI Double Staining 26
18. Intracellular Total ROS Measurements..26
19. Cell Death Rescue Experiments 26
20. Intracellular GPX4 Activity Measurements 27
21. Intracellular Lipid Peroxidation Assays 28
22. Mitochondrial Membrane Potential Measurements 29
23. Lysosomal Membrane Permeabilization Detections 29
24. Hemolysis Analysis..30
25. In Vivo Biodistribution..31
26. In Vivo Antitumor Therapy..32
27. Supplemental References 33

1. Experimental Materials

5,10,15,20-tetrakis(4-formylphenyl)porphyrin and Bodipy-CHO were purchased from Jilin Chinese Academy of Sciences - Yanshen Technology Co., Ltd.

4,4'-disulfanediyldiphenylamine was purchased from Aladdin Reagent (Shanghai) Co., Ltd.

Glutathione (GSH) was purchased from Shanghai Macklin Biochemical Co., Ltd.

5,5',6,6'-tetrachloro-1,1',3,3'-tetraethylbenzimidazolylcarbocyanine iodide (JC-1), 2',7'-dichlorodihydrofluorescein diacetate (DCFH-DA), and 5,5'-Dithiobis-(2-nitrobenzoic acid) (DTNB) were purchased from MedChemExpress (Shanghai, P. R. China).

MitoTracker Deep Red FM, BODIPY581/591 undecanoic acid (C_{11}-BODIPY), and Trypan Blue were purchased from Invitrogen (Thermo Fisher Scientific Inc.).

Acridine Orange (AO), and Giemsa staining solution were purchased from Beijing Solarbio Science & Technology Co., Ltd.

Glutathione peroxidase assay kit, Bradford protein assay kit were purchased from Beyotime (Shanghai, P. R. China).

GSH assay kit was purchased from Nanjing Jiancheng Bioengineering Institute (P. R. China).

3-methyladenine (3-MA) was purchased from TCI (Shanghai) Development Co., Ltd.

Glutathione ethyl ester (GSH-OEt) was purchased from Sigma-Aldrich.

CCK-8 assay kit was purchased from Dojindo (Shanghai, P.R. China).

Ferrostatin-1, liproxstatin-1, and necrostatin-1 were purchased from MedChemExpress (Shanghai, P. R. China).

Paraformaldehyde (4 vol%) fix solution was purchased from Biosharp (Hefei, P. R. China).

Hematoxylin-eosin (H&E) staining kit, terminal deoxynucleotidyl transferase-mediated dUTP nick-end labeling (TUNEL) immunofluorescence assay kit, and Ki67 immunohistochemistry staining kit were purchased from Wuhan Servicebio Technology Co., Ltd.

Trypsin (0.25 wt%) and EDTA (0.02 wt%) in Puck's saline A (trypsin/EDTA solution), Phosphate-buffered saline (PBS), and Dulbecco's phosphate-buffered saline (DPBS) were purchased from Biological Industries USA, Inc.

Certified fetal bovine serum (FBS) was purchased from VivaCell (Shanghai, P. R. China).

Hank's balanced salt solution (HBSS), GlutaMAX (100×), and soybean trypsin inhibitor powder were purchased from Gibco (Thermo Fisher Scientific Inc.).

Dulbecco's modified eagle medium (DMEM) was purchased from HyClone Laboratories, Inc.

Normocin was purchased from Invivogen (San Diego, CA, USA).

Mammary epithelial cell growth medium (MEGM) BulletKit was purchased from Lonza Walkersville, Inc.

Normal saline (NS) was purchased from Shandong Qidu Pharmaceutical Co. Ltd.

2. Cell Culture and Experimental Animals

MCF-7 (human breast adenocarcinoma) cell lines were provided by Institute of Basic Medicine, Shandong Academy of Medical Sciences (Jinan, P. R. China). MCF-10A (human mammary epithelia) cell line was provided by Stem Cell Bank, Chinese Academy of Sciences (Shanghai, P. R. China).

MCF-7 cells were cultured in DMEM supplemented with FBS (10 vol%), human recombinant insulin (10 μg/mL) and Normocin (100 μg/mL) in a 5 vol% CO_2 atmosphere at 37 °C. MCF-10A cells were cultured using MEGM BulletKit supplemented with Normocin (100 μg/mL) in a 5 vol% CO_2 atmosphere at 37 °C. When necessary, trypsin/EDTA solution was used to dissociate cells, and

FBS-containing culture media, or soybean trypsin inhibitor (2.0 mg/mL) was used to discontinue the dissociation.

All animal procedures were reviewed and approved by the Ethics Committee of Shandong Normal University (Jinan, P. R. China), approval number AEECSDNU2022050. All the animal experiments complied with relevant guidelines of the Chinese government and regulations for the care and use of experimental animals. Nude mice (BALB/cJGpt-Foxn1nu/Gpt, aged 4 weeks) were purchased from Hangzhou Ziyuan Laboratory Animal Technology Co., Ltd. The nude mice were housed in a pathogen-free facility and kept in a temperature-controlled room set to light and dark cycle of 12 h each. To establish the MCF-7 xenograft model, MCF-7 cells (10^7 cells) suspended in HBSS (40 μL) were subcutaneously injected into the flanks of each mouse. The length (L) and width (W) of the tumor were determined using digital calipers. The tumor volume (V) was calculated by the formula: $V = 1/2 \times L \times W^2$.

3. Experimental Instrumentations

Fourier transform infrared (FT-IR) spectra were obtained in the 4000~400 cm^{-1} range using a Thermo Scientific Nicolet iS50 FT-IR Spectrometer equipped with a diamond attenuated total reflection (ATR) module. Each spectrum was an average of 16 scans.

Ultraviolet-visible (UV-vis) absorption spectra were recorded on a Shimadzu UV-2700 Double Beam UV-vis Spectrophotometer using 10 mm quartz cuvettes.

Ultraviolet-visible absorption spectra were recorded on a Shimadzu UV-2700 Double Beam UV-Vis Spectrophotometer.

Electron paramagnetic resonance (EPR) spectra were recorded on a Bruker A300 EPR Spectroscopy.

Liquid chromatography-tandem mass spectrometry (LC-MS/MS) analyses were carried out on SCIEX ExionLC AD and QTRAP 6500+ LCMS/MS Systems.

Powder X-ray diffraction (PXRD) patterns were obtained on a Rigaku SmartLab SE X-Ray Powder Diffractometer with Cu Kα line focused radiation (λ = 1.5405 Å) from 2θ = 2.00° up to 30.00° with 0.01° increment.

Nitrogen-adsorption isotherms were measured at 77 K with a Micromeritics ASAP2020 HD88 Surface Area and Porosity Analyser. Before measurement, the samples were degassed a in vacuum at 120 °C for 8 h. The Brunauer-Emmett-Teller (BET) equation was used to calculate the specific surface areas. The pore size distribution was derived from the sorption curve using the non-local density functional theory (NLDFT) model.

Transmission electron microscopy (TEM) images were recorded on a Hitachi HT7700 120 kV Compact-Digital Transmission Electron Microscope. High-angle annular dark field scanning transmission electron microscopy (HAADF-STEM) images and elemental mapping images were recorded using an FEI Talos F200X High-Resolution Scanning Transmission Electron Microscope. To prepare the TEM samples, the nanomaterial was dispersed in methanol by sonication for 5 min and the dispersion was placed on a carbon-coated copper TEM grid (300 mesh) and dried at room temperature.

Hydrodynamic particle size and Zeta potential were measured using Malvern Zetasizer Nano ZS90 System.

Microplate assays were carried out on a Molecular Devices SpectraMax i3x Multi-Mode Microplate Detection System.

Cell counting was performed on a Thermo Fisher Scientific Invitrogen Countess II Automated Cell

Counter equipped with Countess Cell Counting Chamber Slides.

Photomicrographs of biological samples were taken with a Leica DMI3000 B Inverted Fluorescence Microscope with an objective lens (10×, 20×, and 40×).

Laser scanning confocal fluorescence images of cells were captured with a Leica TCS SP8 Confocal Laser Scanning Microscopy equipped with 405, 458, 488, 514, 561, and 633 nm lasers. Glass bottom dishes and 4/8-well chamber slides (Cellvis, Mountain View, CA, USA) were used for cell culture to provide biological replicates of each experiment. Before live cell imaging, the original culture media or DPBS was replaced with HBSS supplemented with HEPES (15 mM) and GlutaMAX to provide better-buffering capacity under normal CO_2 concentration.

For imaging, the scan speed was 400 Hz and transmitted light was used to find the areas of interest to reduce photodamage to the biosample.

4. Statistical Analysis

All results are depicted as means ± SD of at least three biological replicates, as indicated in figure legends. And data were compared with the paired or unpaired two-tailed Student's t-test with or without Welch's correction, two-way ANOVA followed by Šídák's post hoc test, as appropriate. ns, no significance ($p > 0.05$), *$p < 0.05$, **$p < 0.01$.

5. Synthesis of DSPP-COF

A mixture of 5,10,15,20-tetrakis(4-formylphenyl)porphyrin (TFPP) (18.12 mg, 0.025 mmol), 4-(2-(4-aminophenyl)disulfanyl)benzenamine (APDSBA) (12.42 mg, 0.05 mmol) in 1.0 mL *o*-dichlorobenzene and 1.0 mL *n*-BuOH was sonicated for 10 min, followed by slow addition of 0.25 mL of 6 M aqueous acetic acid. Afterward, the tube was flash-frozen at 77 K and degassed by three freeze-pump-thaw cycles, sealed under a vacuum, and heated at 120 ºC for 3 days. After cooling down to room temperature, the resulting precipitate was filtered out, thoroughly washed with *o*-dichlorobenzene, ethanol, and acetone until the filtrate was colorless, and Soxhlet extractions with trichloromethane and dichloromethane for 24 h, respectively. After dried in vacuum at 70 ºC overnight, **DSPP-COF** was obtained as a modena crystalline solid (22.68 mg, 78.9%).

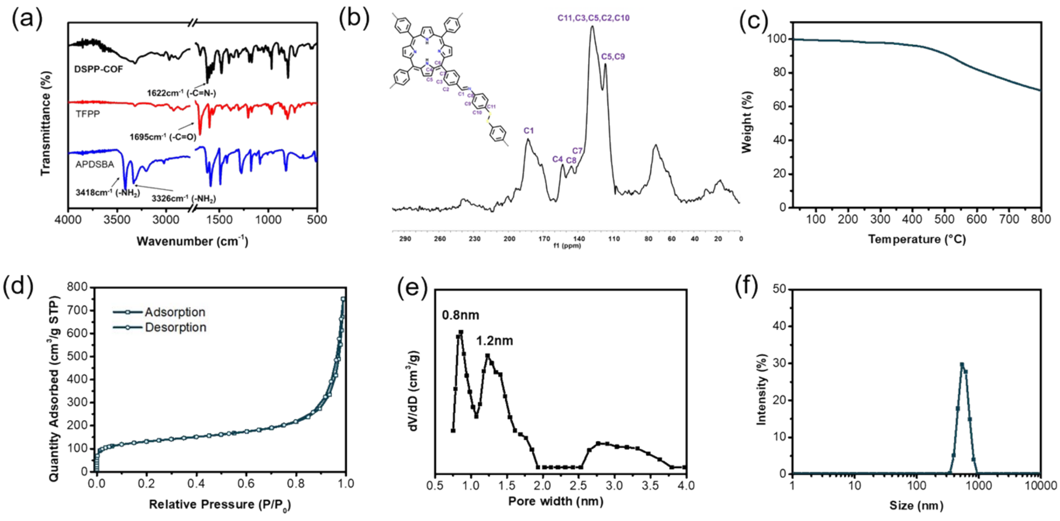

Fig. S1 (a) IR spectra of **DSPP-COF** and its monomers. The peaks at 3416 and 3326 cm^{-1} (for -NH_2) in APDSBA, and 1695 cm^{-1} (for -CHO) in TFPP basically disappeared, and the peaks at 1622 cm^{-1} (for -C=N-) appeared. (b) ^{13}C CP-MAS solid-state NMR spectrum of **DSPP-COF**. C_1 is the carbon atom for the imine bond (183 ppm), and the carbon atoms in both monomers [DFPP (C_2-

C_7), APDSBA (C_8-C_{11})] are represented in **DSPP-COF**. (c) TGA trace of **DSPP-COF**. (d) N_2 adsorption and desorption isotherms of **DSPP-COF**. BET Surface Area of **DSPP-COF** = 477.5 m²/g. (e) Pore size distributions of **DSPP-COF**. The pore size distribution curve based on nonlocal density functional theory (NLDFT) analysis, the pore width of **DSPP-COF** was centered at 0.8 and 1.2 nm. (f) Intensity (%) vs size distribution of **DSPP-COF**.

Table S1 Fractional Atomic Coordinates for the Unit Cell of **DSPP-COF**

DSPP-COF AA-stacking mode and AB-stacking mode

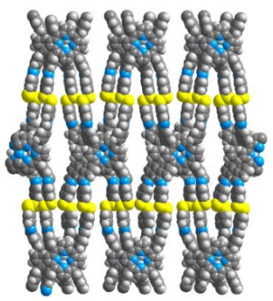<br>AA-stacking mode<br>Space group: *P*1<br>*a* = 20.8379 Å, b = 58.5083 Å, *c* = 3.4124 Å<br>*α* = 90.0521°, *β* = 90.4837°, *γ* = 89.6981°				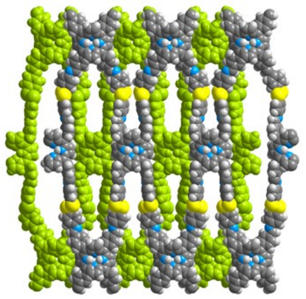<br>AB-stacking mode<br>Space group: *PMN*21<br>*a* = 20.9757 Å, b = 58.1098 Å, *c* = 6.4633 Å<br>*α* = *β* = *γ* = 90°			
	x	y	z		x	y	z
C1	0.03151	0.08175	0.18434	C1	0.47363	0.07962	0.19202
C2	0.05274	0.05991	0.22667	C2	0.41837	0.05706	-0.02335
C3	0.11496	0.05483	0.27591	C3	0.37569	0.05023	-0.1801
C4	0.2053	0.02534	0.28639	C4	0.34424	0.06896	-0.28593
C5	0.13888	0.0323	0.28792	C5	0.38288	0.08652	-0.37627
N6	0.10271	0.01305	0.26731	C6	0.35648	0.10721	-0.42774
C7	0.05754	0.96361	0.19507	C7	0.29083	0.11087	-0.39015
C8	0.11972	0.97198	0.22066	C8	0.25113	0.0926	-0.31579
C9	0.16935	0.95578	0.22095	C9	0.2776	0.0717	-0.26528
C10	0.19096	0.94409	0.10586	C10	0.26643	0.1341	-0.42276
C11	0.23014	0.92531	0.11763	N11	0.20661	0.13909	-0.3826
C12	0.2524	0.91755	0.23932	C12	0.18009	0.16154	-0.40133
C13	0.23782	0.93029	0.35082	C13	0.21854	0.18207	-0.4026
C14	0.19832	0.94889	0.34319	C14	0.19069	0.20352	-0.41749
C15	0.29054	0.89587	0.25118	C15	0.12401	0.20498	-0.43142
S16	0.31168	0.78088	0.32671	C16	0.08569	0.18463	-0.43053
C17	0.44798	0.44657	0.33957	C17	0.11338	0.16314	-0.41304
C18	0.43117	0.47145	0.33574	S18	0.08744	0.23252	-0.47013
C19	0.52813	0.58988	0.28705	C19	0.03636	0.57049	0.18222
C20	0.43745	0.56828	0.28989	C20	0.04067	0.54851	0.09227
C21	0.37175	0.56185	0.28312	C21	0.03159	0.45111	0.04225
C22	0.32747	0.57978	0.26062	C22	0.0768	0.45815	-0.11402
C23	0.31298	0.59672	0.35852	C23	0.11007	0.43925	-0.23225

C24	0.29789	0.61889	0.32373	C24	0.16878	0.43185	-0.16537
C25	0.2907	0.62552	0.19226	C25	0.18721	0.40914	-0.20943
C26	0.29837	0.60843	0.09712	C26	0.14734	0.39356	-0.321
C27	0.31382	0.58618	0.12743	C27	0.09225	0.4018	-0.40712
C28	0.27748	0.64899	0.15252	C28	0.07377	0.42445	-0.36204
N29	0.2702	0.66433	0.24251	C29	0.15884	0.36851	-0.31761
C30	0.26179	0.68819	0.24295	N30	0.11856	0.35314	-0.39487
C31	0.26402	0.70212	0.13142	C31	0.12031	0.32887	-0.35736
C32	0.2543	0.72558	0.14107	C32	0.13484	0.32128	-0.16224
C33	0.24379	0.73631	0.26171	C33	0.13545	0.29775	-0.12592
C34	0.24416	0.72243	0.37294	C34	0.12294	0.28123	-0.28328
C35	0.25149	0.69899	0.36306	C35	0.10577	0.28869	-0.4769
S36	0.22498	0.76654	0.27479	C36	0.10338	0.31242	-0.51199
C37	0.14021	0.99446	0.25156	S37	0.13766	0.25132	-0.22692
C38	0.15457	0.07357	0.30699	C38	0.33739	0.01881	-0.42364
C39	0.14582	0.08553	0.42986	C39	0.3665	0.02683	-0.24525
C40	0.17316	0.1063	0.45355	C40	0.35066	0.94959	0.04362
C41	0.21304	0.11739	0.36347	C41	0.28395	0.94588	0.04216
C42	0.22846	0.10482	0.25126	C42	0.25776	0.92439	-0.02296
C43	0.20117	0.08394	0.22145	C43	0.29754	0.90622	-0.08802
C44	0.23573	0.14038	0.39415	C44	0.3643	0.9104	-0.0988
N45	0.24837	0.15465	0.30102	C45	0.39052	0.93191	-0.03473
C46	0.25743	0.17879	0.30951	C46	0.2686	0.88295	-0.13431
C47	0.24729	0.19143	0.42399	N47	0.30344	0.86469	-0.14145
C48	0.25746	0.21483	0.43049	C48	0.28164	0.84086	-0.16654
C49	0.27707	0.22746	0.32184	C49	0.21633	0.83388	-0.17409
C50	0.28612	0.2151	0.20582	C50	0.19842	0.8104	-0.19443
C51	0.27634	0.19163	0.20075	C51	0.24468	0.79336	-0.20646
S52	0.29649	0.25791	0.33401	C52	0.30987	0.80035	-0.19801
C53	0.28213	0.53266	0.26287	C53	0.32783	0.82383	-0.17761
C54	0.28174	0.50935	0.27608	S54	0.21647	0.76327	-0.24955
C55	0.34617	0.50226	0.30129	C55	0.12797	0.48881	-0.32944
N56	0.3878	0.52073	0.30469	C56	0.13132	0.51264	-0.31423
C57	0.34681	0.53962	0.28108	C57	0.09522	0.51914	-0.13825
C58	0.36719	0.47943	0.32316	N58	0.07104	0.50003	-0.04521
C59	0.31882	0.46233	0.31616	C59	0.09019	0.48146	-0.16181
C60	0.30972	0.44613	0.41953	C60	0.08748	0.54214	-0.06265
C61	0.29197	0.42387	0.39287	C61	0.12944	0.56104	-0.14948
C62	0.27765	0.41652	0.26498	C62	0.18948	0.56625	-0.06606
C63	0.27898	0.43299	0.16553	C63	0.2219	0.58729	-0.10601
C64	0.29641	0.45528	0.18925	C64	0.19512	0.60346	-0.22983
C65	0.26339	0.39301	0.22975	C65	0.13701	0.59749	-0.32536
N66	0.26273	0.37738	0.31917	C66	0.1044	0.57649	-0.28439

C67	0.25358	0.3536	0.30334	C67	0.22544	0.62703	-0.23552
C68	0.24275	0.34255	0.18334	N68	0.21068	0.64461	-0.34826
C69	0.23292	0.31907	0.17622	C69	0.23412	0.66929	-0.3051
C70	0.23307	0.30516	0.28795	C70	0.22552	0.68607	-0.45071
C71	0.24538	0.31618	0.40689	C71	0.2439	0.70925	-0.40892
C72	0.25502	0.33958	0.41424	C72	0.27049	0.71592	-0.21956
S73	0.21276	0.27471	0.28031	C73	0.27954	0.69911	-0.07534
C74	0.9808	0.93781	0.14567	C74	0.26153	0.67599	-0.11695
C75	0.96749	0.07995	0.15592	S75	0.30038	0.74528	-0.16411
C76	0.94985	0.05704	0.18259	H76	0.43442	0.08481	-0.39326
C77	0.88446	0.04997	0.18955	H77	0.38804	0.12081	-0.48832
C78	0.79783	0.01925	0.2126	H78	0.2002	0.09484	-0.28889
C79	0.86141	0.02723	0.18353	H79	0.24675	0.05837	-0.19698
N80	0.8972	0.0079	0.16356	H80	0.30024	0.14704	-0.48237
C81	0.04396	0.93946	0.15864	H81	0.26996	0.1818	-0.38957
N82	0.00232	0.97643	0.20528	H82	0.22153	0.21891	-0.42316
C83	0.95437	0.96088	0.1726	H83	0.03424	0.18543	-0.44265
C84	0.88833	0.9661	0.17595	H84	0.08282	0.14764	-0.41149
C85	0.84465	0.94766	0.18955	H85	0.06825	0.58533	0.15627
C86	0.85318	0.93228	0.29742	H86	0.19684	0.44278	-0.06128
C87	0.81284	0.91422	0.3153	H87	0.23019	0.40326	-0.14303
C88	0.76202	0.90923	0.22925	H88	0.06056	0.39027	-0.49402
C89	0.75002	0.92492	0.12804	H89	0.02728	0.42962	-0.40715
C90	0.78928	0.9434	0.107	H90	0.19859	0.36305	-0.23084
C91	0.72559	0.88832	0.24424	H91	0.14513	0.33354	-0.03691
N92	0.74441	0.8741	0.33385	H92	0.14683	0.29234	0.0256
C93	0.74168	0.85006	0.33693	H93	0.09621	0.27653	-0.60305
C94	0.72616	0.83659	0.22766	H94	0.09066	0.31797	-0.66198
C95	0.72568	0.81293	0.23411	H95	0.31866	0.02905	-0.54156
C96	0.73929	0.80152	0.35137	H96	0.25228	0.95913	0.10195
C97	0.75594	0.81499	0.46032	H97	0.20633	0.92163	-0.0127
C98	0.75747	0.83867	0.45314	H98	0.39632	0.89687	-0.15094
S99	0.73122	0.7707	0.36083	H99	0.44227	0.93439	-0.03595
C100	0.51192	0.44522	0.33841	H100	0.21718	0.88144	-0.14833
C101	0.53473	0.46911	0.32698	H101	0.17867	0.84622	-0.16413
C102	0.46238	0.59124	0.28507	H102	0.14799	0.80546	-0.20214
C103	0.54554	0.56624	0.29154	H103	0.34754	0.78805	-0.21225
C104	0.60891	0.55777	0.28846	H104	0.37827	0.82881	-0.17151
C105	0.6596	0.57429	0.283	H105	0.15107	0.47812	-0.44927
C106	0.69583	0.5819	0.39602	H106	0.15731	0.52389	-0.42052
C107	0.72368	0.60328	0.40194	H107	0.20921	0.55473	0.04066
C108	0.72342	0.6187	0.29613	H108	0.26664	0.59143	-0.03208
C109	0.69515	0.6104	0.18064	H109	0.11494	0.60958	-0.4214

C110	0.66694	0.58913	0.17178	H110	0.05763	0.57294	-0.34719
C111	0.75117	0.64161	0.31135	H111	0.20369	0.63454	-0.361
N112	0.74676	0.65747	0.22082	H112	0.20489	0.68114	-0.59703
C113	0.7658	0.68107	0.22918	H113	0.2385	0.72182	-0.52541
C114	0.76139	0.6959	0.12136	H114	0.29994	0.704	0.07099
C115	0.77621	0.71893	0.13202	H115	0.26798	0.66345	-0.00098
C116	0.79516	0.72852	0.25194	C116	0.98028	0.43959	0.12388
C117	0.80229	0.71358	0.35811	H117	0.96346	0.42457	0.2069
C118	0.78898	0.69047	0.34664	H118	0.38894	0.09277	0.05739
S119	0.81267	0.75877	0.26984	C119	0.42333	0.94616	0.41729
C120	0.86248	0.98848	0.17996	C120	0.42156	0.96644	0.31199
C121	0.83949	0.06829	0.20759	C121	0.37942	0.97032	0.15353
C122	0.84281	0.08256	0.32197	C122	0.33841	0.99868	-0.0853
C123	0.81708	0.10427	0.32579	C123	0.36818	0.99292	0.09183
C124	0.78374	0.11414	0.21963	N124	0.38414	1.01242	0.20212
C125	0.77171	0.09907	0.11466	H125	0.39008	0.93144	0.40837
C126	0.79647	0.07712	0.1087	H126	0.32025	0.98699	-0.20495
C127	0.76145	0.13799	0.21043	H127	0.40166	1.01324	0.35033
N128	0.77313	0.15329	0.30116	H128	0.54527	0.51114	0.77354
C129	0.76092	0.17741	0.2936	C129	0.06716	0.06378	0.6642
C130	0.75779	0.19117	0.40627	C130	0.07014	0.04346	0.54519
C131	0.74491	0.2145	0.4009	C131	0.11364	0.03962	0.37562
C132	0.73518	0.22597	0.28193	C132	0.15596	0.01177	0.10547
C133	0.73927	0.21244	0.16951	C133	0.12545	0.01709	0.29457
C134	0.75183	0.18913	0.17499	N134	-0.11005	0.00252	0.39526
S135	0.71273	0.25636	0.27201	H135	0.09956	0.07871	0.65644
C136	0.6885	0.52635	0.27401	H136	0.17367	0.02364	-0.01027
C137	0.68588	0.50313	0.26302	H137	-0.09218	0.00327	0.54752
C138	0.62037	0.49707	0.2731	H138	0.10763	0.91729	0.88338
N139	0.57959	0.51634	0.285	C139	0.07726	0.95284	0.83426
C140	0.62463	0.53437	0.28308	C140	0.11981	0.95959	0.66999
C141	0.59837	0.4745	0.29323	C141	0.14916	0.94065	0.53985
C142	0.64436	0.45645	0.28741	C142	0.10815	0.9245	0.44031
C143	0.66706	0.44515	0.4043	C143	0.13085	0.90342	0.36302
C144	0.69688	0.42422	0.39999	C144	0.19498	0.89781	0.38548
C145	0.70836	0.4126	0.28258	C145	0.23744	0.91461	0.47143
C146	0.69047	0.42401	0.16651	C146	0.21473	0.93604	0.54641
C147	0.66162	0.4452	0.16715	C147	0.21387	0.87397	0.32717
C148	0.73774	0.39001	0.29291	N148	0.2686	0.86578	0.37425
N149	0.73462	0.37555	0.19642	C149	0.28499	0.84184	0.343
C150	0.75045	0.3519	0.20051	C150	0.23736	0.82403	0.33094
C151	0.75085	0.33915	0.31658	C151	0.25403	0.80095	0.31876
C152	0.76448	0.31589	0.31948	C152	0.31823	0.79484	0.31997

C153	0.77486	0.30334	0.20517	C153	0.3664	0.8124	0.3296
C154	0.77232	0.31579	0.08784	C154	0.34971	0.83575	0.34123
C155	0.76094	0.33927	0.08584	S155	0.33253	0.76422	0.34054
S156	0.79555	0.27279	0.21235	C156	0.46918	0.44009	0.60297
N157	0.48536	0.48479	0.33438	C157	0.46515	0.46203	0.52809
N158	0.48961	0.55409	0.293	C158	0.53373	0.5824	0.73845
N159	1.0037	0.04401	0.21868	C159	0.47535	0.55935	0.53769
H160	0.06066	0.09708	0.18094	C160	0.43021	0.55227	0.38905
H161	0.24761	0.0363	0.2914	C161	0.4008	0.5713	0.28259
H162	0.17525	0.95	0.00904	C162	0.34496	0.58125	0.36932
H163	0.24486	0.91619	0.02938	C163	0.3331	0.60445	0.33646
H164	0.25588	0.92436	0.44645	C164	0.37685	0.61798	0.21734
H165	0.18521	0.9579	0.43237	C165	0.42877	0.60721	0.11272
H166	0.41186	0.43331	0.33885	C166	0.4405	0.58408	0.14575
H167	0.56134	0.60423	0.28546	C167	0.37207	0.64345	0.22579
H168	0.32466	0.59303	0.46103	N168	0.41592	0.65672	0.13677
H169	0.29476	0.63199	0.40086	C169	0.41936	0.68153	0.15892
H170	0.29299	0.61284	-0.00623	C170	0.39956	0.6932	0.34394
H171	0.32717	0.57424	0.04904	C171	0.40391	0.71734	0.3611
H172	0.27205	0.69436	0.03578	C172	0.42734	0.73026	0.19324
H173	0.25347	0.73597	0.05216	C173	0.45007	0.71859	0.01283
H174	0.23723	0.73004	0.46961	C174	0.44637	0.69441	-0.00386
H175	0.24941	0.68861	0.45202	S175	0.42895	0.7618	0.20456
H176	0.11417	0.07782	0.50295	C176	0.15773	0.99038	0.43241
H177	0.16293	0.11484	0.54623	C177	0.1284	0.98288	0.61473
H178	0.26374	0.1112	0.18199	C178	0.14536	0.06025	0.27855
H179	0.21327	0.07542	0.12943	C179	0.21236	0.06299	0.26899
H180	0.2331	0.18269	0.51343	C180	0.24165	0.08405	0.21379
H181	0.25008	0.22306	0.52568	C181	0.2048	0.10278	0.16581
H182	0.30272	0.22424	0.11853	C182	0.13773	0.09964	0.16384
H183	0.28673	0.18237	0.11022	C183	0.10835	0.07853	0.21884
H184	0.24014	0.54369	0.2453	C184	0.23729	0.12542	0.12544
H185	0.24011	0.498	0.26846	N185	0.20486	0.1442	0.12351
H186	0.32835	0.45	0.51768	C186	0.23026	0.16752	0.10116
H187	0.29213	0.41143	0.47323	C187	0.29622	0.17301	0.0675
H188	0.26822	0.42805	0.06414	C188	0.31774	0.19606	0.04999
H189	0.30539	0.46691	0.10719	C189	0.27456	0.21407	0.06932
H190	0.24224	0.35255	0.09312	C190	0.20885	0.20862	0.10136
H191	0.22142	0.31148	0.08138	C191	0.18707	0.18556	0.11556
H192	0.24665	0.30656	0.49839	S192	0.30523	0.24354	0.02826
H193	0.26404	0.34746	0.50959	C193	0.37924	0.52155	0.16669
H194	0.94976	0.92388	0.11825	C194	0.37573	0.49772	0.16706
H195	0.93514	0.09371	0.12628	C195	0.41092	0.49127	0.33076

H196	0.75656	0.02961	0.2405	N196	0.43452	0.5104	0.43135
H197	0.08213	0.92697	0.14788	C197	0.41619	0.52895	0.33076
H198	0.89326	0.93491	0.36604	C198	0.4189	0.46827	0.38816
H199	0.8233	0.9029	0.39761	C199	0.37872	0.44912	0.29537
H200	0.70962	0.92198	0.06059	C200	0.31784	0.44359	0.38471
H201	0.77877	0.95469	0.02399	C201	0.28803	0.42197	0.34225
H202	0.71563	0.84474	0.1335	C202	0.31833	0.40556	0.21035
H203	0.71382	0.80338	0.14494	C203	0.37695	0.41189	0.10766
H204	0.76649	0.80704	0.55548	C204	0.40688	0.43345	0.15075
H205	0.7707	0.84888	0.5396	C205	0.29155	0.38144	0.204
H206	0.54469	0.43077	0.33761	N206	0.30939	0.36381	0.07989
H207	0.4355	0.60724	0.27731	C207	0.29222	0.33875	0.12107
H208	0.69407	0.5717	0.48561	C208	0.30049	0.32194	-0.03815
H209	0.74564	0.60869	0.49527	C209	0.28734	0.2985	-0.0019
H210	0.69351	0.62168	0.09499	C210	0.26677	0.29148	0.19628
H211	0.64134	0.58458	0.08173	C211	0.25886	0.30837	0.35519
H212	0.74662	0.68933	0.02534	C212	0.27114	0.33179	0.31846
H213	0.7718	0.72953	0.04388	S213	0.24244	0.2616	0.25177
H214	0.81572	0.72027	0.45445	H214	0.05732	0.92759	0.4359
H215	0.79757	0.67979	0.43315	H215	0.09719	0.89093	0.29553
H216	0.87105	0.07593	0.40471	H216	0.28743	0.91088	0.48648
H217	0.8233	0.1142	0.4159	H217	0.24732	0.94833	0.62134
H218	0.74255	0.10431	0.02986	H218	0.17879	0.86297	0.24942
H219	0.7876	0.06659	0.02172	H219	0.18686	0.82765	0.34013
H220	0.76368	0.18324	0.5028	H220	0.21626	0.78764	0.31557
H221	0.73969	0.22396	0.49299	H221	0.41631	0.80823	0.3374
H222	0.73071	0.22023	0.07363	H222	0.38717	0.84908	0.35382
H223	0.75458	0.17982	0.08217	H223	0.43724	0.42526	0.576
H224	0.73179	0.53687	0.2785	H224	0.55124	0.59762	0.8228
H225	0.72707	0.4915	0.25941	H225	0.31451	0.57194	0.47982
H226	0.65648	0.45294	0.49912	H226	0.29234	0.61227	0.41665
H227	0.71151	0.41647	0.49347	H227	0.46323	0.6171	0.01925
H228	0.69891	0.41593	0.07148	H228	0.48496	0.57692	0.08624
H229	0.6463	0.45298	0.07468	H229	0.33303	0.651	0.31799
H230	0.7394	0.34735	0.40966	H230	0.38156	0.68371	0.47608
H231	0.76716	0.30728	0.41461	H231	0.38908	0.72587	0.50564
H232	0.7813	0.30701	-0.00487	H232	0.46937	0.72825	-0.11679
H233	0.76048	0.34815	-0.00829	H233	0.46336	0.68572	-0.14556
H234	0.4873	0.5006	0.29733	H234	0.17644	0.97989	0.30392
H235	0.25638	0.38915	0.12576	H235	0.24183	0.04927	0.31488
H236	0.7577	0.38554	0.38953	H236	0.29314	0.08601	0.21841
H237	0.69144	0.88383	0.16593	H237	0.10794	0.11362	0.12491
H238	0.73754	0.14243	0.11789	H238	0.05642	0.07678	0.22404

H239	0.23419	0.14543	0.4975	H239	0.28911	0.12607	0.11089
H240	0.27623	0.65287	0.0477	H240	0.33156	0.15984	0.05209
N241	0.26377	0.88364	0.36359	H241	0.36847	0.19988	0.02124
H242	0.05513	0.0127	0.24935	H242	0.17403	0.22201	0.11126
C243	0.20602	0.0021	0.26224	H243	0.13615	0.18171	0.13915
C244	-0.79851	0.00425	0.21206	H244	0.35698	0.53221	0.05853
H245	0.48627	0.53703	0.30037	H245	0.35044	0.48644	0.05875
H246	0.94402	0.00684	0.14412	H246	0.29561	0.45528	0.49862
C247	-0.13122	0.07491	0.76503	H247	0.24261	0.41762	0.42066
C248	-0.10658	0.05691	0.68821	H248	0.40171	0.39964	0.0052
C249	-0.04229	0.05445	0.65011	H249	0.45436	0.43723	0.08351
C250	0.04971	0.02628	0.6123	H250	0.31382	0.37397	0.06806
C251	-0.01623	0.03282	0.62206	H251	0.31621	0.32708	-0.1915
N252	-0.05045	0.01283	0.63372	H252	0.29122	0.28617	-0.13059
C253	-0.0928	0.96228	0.66016	H253	0.243	0.30337	0.50866
C254	-0.02994	0.97135	0.66725	H254	0.26514	0.34438	0.44475
C255	0.01872	0.9544	0.67342	C255	1.02676	0.94121	0.91794
C256	0.05462	0.94743	0.56053	H256	0.9663	0.51065	0.20161
C257	0.08515	0.92651	0.5562	C257	1.01771	0.07328	0.66004
C258	0.08785	0.9115	0.66372	C258	0.98891	0.05197	0.61172
C259	0.05835	0.91921	0.77872	C259	0.92521	0.04539	0.67088
C260	0.02499	0.93956	0.78458	C260	0.84102	0.01357	0.68354
C261	0.11817	0.88904	0.65009	C261	0.90418	0.02188	0.6691
N262	0.12147	0.87484	0.74823	N262	0.94391	0.00388	0.65391
C263	0.12789	0.85059	0.74446	H263	0.99923	0.0866	0.76567
C264	0.14323	0.8378	0.85712	H264	0.79817	0.02361	0.6967
C265	0.14424	0.81406	0.8563	H265	0.99386	0.00501	0.657
C266	0.12938	0.80153	0.74295	C266	0.07554	0.93126	0.91902
C267	0.11718	0.81419	0.62922	H267	0.04847	0.49965	0.10049
C268	0.11721	0.83793	0.62965	C268	0.99605	0.96183	0.90312
S269	0.13122	0.77031	0.7415	C269	0.93146	0.96541	0.97043
C270	0.27734	0.44627	0.77449	C270	0.88806	0.94426	0.98677
C271	0.26533	0.47025	0.73173	C271	0.89089	0.92739	0.82512
C272	0.37886	0.58559	0.76623	C272	0.86456	0.90529	0.85199
C273	0.28693	0.56538	0.72857	C273	0.82849	0.89997	1.03206
C274	0.22151	0.55999	0.70376	C274	0.82046	0.91728	1.18844
C275	0.17573	0.57794	0.70423	C275	0.85128	0.93918	1.16794
C276	0.1475	0.58695	0.82229	C276	0.80188	0.87607	1.05023
C277	0.11391	0.60742	0.82286	N277	0.74182	0.87182	1.10395
C278	0.10048	0.62012	0.70881	C278	0.71015	0.84921	1.0977
C279	0.12156	0.61002	0.59229	C279	0.74413	0.82861	1.09978
C280	0.1573	0.59026	0.58719	C280	0.71149	0.80706	1.09148
C281	0.06739	0.64229	0.69627	C281	0.64455	0.80557	1.07716

N282	0.05799	0.65626	0.79417	C282	0.61063	0.82598	1.07459
C283	0.0479	0.68057	0.78353	C283	0.643	0.84759	1.08829
C284	0.05326	0.69301	0.66623	S284	0.60272	0.77771	1.03464
C285	0.05009	0.71679	0.66139	C285	0.52265	0.4404	0.71784
C286	0.04009	0.72976	0.77425	C286	0.54965	0.46258	0.71197
C287	0.03012	0.71753	0.89002	C287	0.48291	0.58189	0.61398
C288	0.03402	0.69383	0.89453	C288	0.55596	0.56013	0.73748
S289	0.03631	0.76095	0.7675	C289	0.60985	0.55373	0.84617
C290	-0.01099	0.99433	0.64559	C290	0.64484	0.57289	0.95901
C291	-0.00497	0.07502	0.65107	C291	0.62802	0.57731	1.16861
C292	0.05632	0.07834	0.71494	C292	0.63474	0.5997	1.25587
C293	0.08321	0.09985	0.7256	C293	0.65706	0.61805	1.13379
C294	0.05411	0.11977	0.67404	C294	0.68068	0.61313	0.93102
C295	-0.00481	0.11652	0.61011	C295	0.67586	0.59059	0.84563
C296	-0.03247	0.09543	0.59533	C296	0.65088	0.64212	1.21554
C297	0.08232	0.14262	0.68539	N297	0.64414	0.65893	1.09106
N298	0.06101	0.15934	0.61256	C298	0.63422	0.68294	1.14696
C299	0.0728	0.18304	0.62728	C299	0.63518	0.69186	1.35195
C300	0.0714	0.19347	0.7505	C300	0.63149	0.71563	1.39412
C301	0.08157	0.21669	0.76722	C301	0.62728	0.73104	1.23445
C302	0.09313	0.23112	0.66127	C302	0.62246	0.7221	1.03115
C303	0.09423	0.22084	0.53756	C303	0.62554	0.69824	0.98875
C304	0.08443	0.19751	0.52064	S304	0.63914	0.76176	1.30115
S305	0.1111	0.26134	0.68658	C305	0.84281	0.99342	1.01274
C306	0.13059	0.53226	0.66845	C306	0.90706	0.98792	0.99213
C307	0.12692	0.50919	0.64767	C307	0.87961	0.06455	0.71045
C308	0.19093	0.50075	0.65183	C308	0.84788	0.06808	0.90397
N309	0.23634	0.51853	0.66822	C309	0.81651	0.08879	0.95109
C310	0.19701	0.53787	0.68038	C310	0.81829	0.10649	0.80805
C311	0.20747	0.47756	0.67221	C311	0.84736	0.10251	0.61172
C312	0.15964	0.46061	0.64825	C312	0.87661	0.0816	0.56226
C313	0.12857	0.4488	0.75366	C313	0.78776	0.12862	0.8624
C314	0.10616	0.42678	0.73803	N314	0.81149	0.14818	0.79667
C315	0.10737	0.41521	0.61792	C315	0.78181	0.17009	0.83665
C316	0.13188	0.42738	0.51148	C316	0.71468	0.17208	0.84139
C317	0.15615	0.44915	0.52339	C317	0.68743	0.19386	0.858
C318	0.0861	0.3917	0.60029	C318	0.72633	0.21403	0.87378
N319	0.0726	0.37913	0.70106	C319	0.79327	0.212	0.87392
C320	0.06131	0.35524	0.70491	C320	0.82065	0.19025	0.85185
C321	0.06477	0.3412	0.59398	S321	0.6889	0.24195	0.85676
C322	0.05313	0.31789	0.59768	C322	0.6885	0.52417	0.92134
C323	0.03935	0.30686	0.71529	C323	0.68847	0.5003	0.90597
C324	0.03636	0.32071	0.82735	C324	0.62925	0.4929	0.83353

C325	0.0473	0.34411	0.82218	N325	0.59359	0.51152	0.80576
S326	0.0223	0.27626	0.71749	C326	0.62964	0.53067	0.85552
C327	0.82736	0.93781	0.61653	C327	0.6083	0.46949	0.80301
C328	0.80395	0.07413	0.75301	C328	0.64942	0.4504	0.86097
C329	0.79065	0.05528	0.66765	C329	0.64733	0.44293	1.06489
C330	0.72996	0.04968	0.61816	C330	0.66761	0.42081	1.10697
C331	0.6463	0.01939	0.67475	C331	0.68897	0.40565	0.94611
C332	0.70799	0.02732	0.62993	C332	0.69551	0.4139	0.74424
N333	0.74866	0.00842	0.64115	C333	0.67659	0.43611	0.70297
C334	-0.10752	0.93914	0.62983	C334	0.6994	0.3811	0.99184
N335	-0.14855	0.9754	0.66691	N335	0.70163	0.363	0.85849
C336	0.80209	0.9599	0.64021	C336	0.70809	0.33804	0.91521
C337	0.73715	0.96637	0.64369	C337	0.70285	0.32059	0.76054
C338	0.6916	0.94843	0.63399	C338	0.7115	0.29742	0.80415
C339	0.64332	0.94767	0.53198	C339	0.72545	0.29135	1.00475
C340	0.60776	0.92825	0.5093	C340	0.72898	0.30879	1.16039
C341	0.61335	0.90861	0.5882	C341	0.72037	0.33196	1.11691
C342	0.65717	0.91001	0.69304	S342	0.7494	0.26236	1.06435
C343	0.69471	0.92875	0.71722	H343	0.91782	0.93081	0.68218
C344	0.57625	0.88793	0.56131	H344	0.87186	0.8923	0.72966
N345	0.5849	0.86988	0.63373	H345	0.79348	0.91348	1.3299
C346	0.55999	0.84728	0.62709	H346	0.84856	0.95173	1.29588
C347	0.52946	0.8378	0.51662	H347	0.83102	0.86236	0.984
C348	0.50787	0.81535	0.51468	H348	0.7957	0.82899	1.11076
C349	0.51628	0.80076	0.62326	H349	0.73874	0.79154	1.0911
C350	0.5458	0.81047	0.73382	H350	0.55907	0.82514	1.06022
C351	0.56874	0.83258	0.73375	H351	0.61584	0.86314	1.0853
S352	0.48932	0.77082	0.63086	H352	0.53969	0.42586	0.79713
C353	0.33965	0.4449	0.80005	H353	0.45493	0.59662	0.58308
C354	0.36708	0.46774	0.77093	H354	0.6034	0.56406	1.2584
C355	0.31321	0.58762	0.75427	H355	0.61719	0.60303	1.41352
C356	0.39487	0.56182	0.74877	H356	0.69935	0.6269	0.83503
C357	0.45739	0.55216	0.74651	H357	0.68871	0.58763	0.68327
C358	0.50888	0.56745	0.76513	H358	0.64685	0.64498	1.38114
C359	0.52085	0.57725	0.89194	H359	0.64121	0.68092	1.48004
C360	0.54368	0.59922	0.90397	H360	0.63503	0.72219	1.5519
C361	0.55992	0.61245	0.79465	H361	0.61955	0.73333	0.90333
C362	0.55694	0.60171	0.67154	H362	0.62384	0.69174	0.83039
C363	0.53493	0.5797	0.65624	H363	0.80154	0.98155	1.0149
C364	0.57583	0.63646	0.81306	H364	0.84994	0.05536	1.02126
N365	0.57392	0.64981	0.71241	H365	0.79331	0.09142	1.10258
C366	0.56665	0.67389	0.70479	H366	0.84767	0.1156	0.4969
C367	0.53626	0.68338	0.59469	H367	0.90031	0.07922	0.41124

C368	0.53411	0.70684	0.57411	H368	0.74607	0.12837	0.96431
C369	0.56157	0.72231	0.66349	H369	0.68346	0.15696	0.82034
C370	0.58707	0.71282	0.77915	H370	0.63585	0.19504	0.85211
C371	0.59023	0.68929	0.79839	H371	0.82476	0.22713	0.88298
S372	0.57521	0.7527	0.62243	H372	0.8724	0.1891	0.84436
C373	0.71383	0.98894	0.65517	H373	0.72765	0.53544	0.97256
C374	0.69127	0.06951	0.57914	H374	0.72746	0.48964	0.94483
C375	0.72531	0.08939	0.53765	H375	0.62556	0.45347	1.18866
C376	0.69579	0.10958	0.50205	H376	0.66239	0.41492	1.26347
C377	0.62911	0.11231	0.50698	H377	0.71218	0.40279	0.6166
C378	0.59438	0.09232	0.53488	H378	0.67808	0.44145	0.54447
C379	0.62321	0.07195	0.56977	H379	0.69985	0.37288	0.84336
C380	0.6	0.13457	0.48162	H380	0.69363	0.3251	0.60442
N381	0.57931	0.1467	0.57978	H381	0.71056	0.28461	0.67871
C382	0.56353	0.17044	0.58601	H382	0.73994	0.30445	1.31541
C383	0.55347	0.1815	0.70562	H383	0.72477	0.34509	1.23919
C384	0.541	0.20481	0.7154	H384	1.00962	0.92618	1.00063
C385	0.53766	0.21893	0.60508	C385	0.02557	0.4288	0.11162
C386	0.54809	0.20806	0.4847	H386	0.05466	0.41421	0.0668
C387	0.56	0.18467	0.47578	H387	0.49075	0.09454	0.28183
S388	0.51447	0.24929	0.61696	C388	0.42033	0.07864	0.07601
C389	0.53705	0.51966	0.72673	C389	0.50378	0.05886	0.15595
C390	0.53119	0.49639	0.7215	C390	0.56797	0.05505	0.20551
C391	0.46432	0.49187	0.72481	C391	0.61186	0.07604	0.22286
N392	0.42726	0.51206	0.72329	C392	0.61058	0.09247	0.06916
C393	0.47404	0.52906	0.72897	C393	0.63658	0.11464	0.10174
C394	0.43353	0.47097	0.75145	C394	0.67062	0.12047	0.27876
C395	0.47348	0.45147	0.76009	C395	0.67719	0.10359	0.42599
C396	0.50651	0.44454	0.87805	C396	0.6468	0.08159	0.40034
C397	0.52888	0.4225	0.89298	C397	0.69636	0.14448	0.30476
C398	0.52399	0.40625	0.79175	N398	0.75658	0.14897	0.34084
C399	0.4938	0.41312	0.67506	C399	0.7877	0.17166	0.33879
C400	0.46921	0.4346	0.65873	C400	0.75332	0.19216	0.36517
C401	0.54729	0.38298	0.80614	C401	0.7855	0.21379	0.36164
N402	0.53846	0.3689	0.70941	C402	0.85239	0.21547	0.32816
C403	0.54927	0.3452	0.70176	C403	0.88661	0.19515	0.29987
C404	0.5551	0.33106	0.81157	C404	0.85474	0.17346	0.30892
C405	0.56714	0.30778	0.80097	S405	0.89399	0.24343	0.29575
C406	0.57403	0.29721	0.67833	C406	0.98811	0.58143	0.18425
C407	0.56663	0.31144	0.56844	C407	0.96129	0.55922	0.17121
C408	0.55396	0.33474	0.57924	C408	0.95661	0.46169	0.13773
S409	0.59794	0.26694	0.6625	C409	0.90197	0.46805	0.26211
N410	0.31974	0.4835	0.74221	C410	0.86628	0.44887	0.3687

N411	0.33816	0.55069	0.72985	C411	0.88199	0.44418	0.57103
N412	0.84506	0.04375	0.63456	C412	0.87322	0.4218	0.64389
H413	-0.10268	0.08755	0.81679	C413	0.84991	0.40372	0.51413
H414	0.09056	0.03774	0.60535	C414	0.82795	0.40895	0.31986
H415	0.05105	0.95812	0.47284	C415	0.83508	0.43146	0.24911
H416	0.10676	0.92121	0.46317	C416	0.8533	0.37955	0.57799
H417	0.05737	0.90817	0.86518	N417	0.85808	0.36263	0.44029
H418	-0.00274	0.94405	0.87111	C418	0.86536	0.33847	0.47722
H419	0.15482	0.84663	0.94893	C419	0.86255	0.32952	0.67635
H420	0.15636	0.80488	0.9466	C420	0.86432	0.30569	0.70129
H421	0.10697	0.80594	0.53519	C421	0.86837	0.29026	0.52994
H422	0.1067	0.8469	0.53751	C422	0.87477	0.29918	0.33157
H423	0.24005	0.43342	0.7818	C423	0.87353	0.32309	0.30628
H424	0.41244	0.59973	0.78382	S424	0.8541	0.25962	0.57779
H425	0.15875	0.57833	0.91518	C425	0.65574	0.02675	0.20732
H426	0.09778	0.61416	0.91709	C426	0.5917	0.03247	0.20671
H427	0.11081	0.6187	0.49957	C427	0.61701	0.95571	0.54289
H428	0.17428	0.58451	0.49128	C428	0.65116	0.95253	0.72107
H429	0.0622	0.68441	0.57339	C429	0.68358	0.93199	0.74283
H430	0.05622	0.72558	0.56751	C430	0.68046	0.91411	0.59008
H431	0.01981	0.72703	0.97991	C431	0.64875	0.91769	0.40865
H432	0.02554	0.68511	0.98747	C432	0.61847	0.93843	0.38372
H433	0.08227	0.06361	0.75763	C433	0.71218	0.89216	0.618
H434	0.13038	0.10098	0.77409	N434	0.68724	0.87243	0.55383
H435	-0.03152	0.1312	0.5722	C435	0.71705	0.85052	0.56867
H436	-0.07942	0.09476	0.54587	C436	0.78414	0.84845	0.55048
H437	0.06186	0.18308	0.83713	C437	0.81119	0.82664	0.54485
H438	0.08084	0.22387	0.86583	C438	0.77215	0.80653	0.56016
H439	0.10359	0.23157	0.45181	C439	0.70529	0.80865	0.5824
H440	0.08826	0.19006	0.42233	C440	0.6781	0.83042	0.58316
H441	0.08257	0.4991	0.6409	S441	0.8093	0.77855	0.51514
H442	0.13257	0.45561	0.85356	C442	0.82235	0.49752	0.37329
H443	0.08748	0.41816	0.82489	C443	0.82224	0.52138	0.37401
H444	0.13388	0.41945	0.41412	C444	0.88177	0.52884	0.29298
H445	0.18144	0.45619	0.43863	N445	0.91779	0.51024	0.24451
H446	0.07641	0.34876	0.49972	C446	0.88166	0.49107	0.28985
H447	0.05532	0.30804	0.50653	C447	0.90264	0.55226	0.2735
H448	0.02395	0.31317	0.92138	C448	0.86116	0.57127	0.35569
H449	0.04296	0.35438	0.91124	C449	0.86257	0.57868	0.56445
H450	0.79931	0.92258	0.59404	C450	0.84059	0.60057	0.62796
H451	0.76901	0.08568	0.79619	C451	0.81811	0.61553	0.48386
H452	0.60536	0.02983	0.70293	C452	0.81264	0.60733	0.27754
H453	-0.07289	0.92527	0.61756	C453	0.83335	0.58536	0.21508

H454	0.64043	0.96216	0.46386	C454	0.80528	0.63989	0.55052
H455	0.57565	0.92803	0.42388	N455	0.80068	0.65792	0.43092
H456	0.66336	0.89554	0.75906	C456	0.792	0.68275	0.50724
H457	0.73057	0.92822	0.7966	C457	0.79567	0.70023	0.36341
H458	0.52382	0.84874	0.43032	C458	0.78569	0.7233	0.42601
H459	0.48347	0.80978	0.42463	C459	0.77197	0.72923	0.63503
H460	0.55023	0.80046	0.82456	C460	0.76947	0.71176	0.77924
H461	0.59257	0.83921	0.82206	C461	0.7795	0.68869	0.71678
H462	0.36926	0.43084	0.83348	S462	0.74756	0.75812	0.72203
H463	0.28642	0.6036	0.76169	H463	0.58487	0.08866	-0.07055
H464	0.50052	0.56858	0.97731	H464	0.63036	0.12732	-0.01332
H465	0.54407	0.60722	1.00101	H465	0.70248	0.10781	0.56526
H466	0.56729	0.6113	0.58179	H466	0.64816	0.0694	0.52229
H467	0.52594	0.57319	0.55729	H467	0.66651	0.15803	0.25686
H468	0.51613	0.67193	0.51931	H468	0.7018	0.19165	0.39206
H469	0.51354	0.7132	0.48251	H469	0.7579	0.22922	0.3806
H470	0.61082	0.72405	0.851	H470	0.93802	0.19611	0.26907
H471	0.61477	0.68267	0.88525	H471	0.88218	0.15799	0.28662
H472	0.77783	0.08862	0.53968	H472	0.97085	0.59598	0.27768
H473	0.72761	0.12357	0.47458	H473	0.90701	0.45718	0.66467
H474	0.54174	0.09266	0.5297	H474	0.88978	0.41824	0.79515
H475	0.59043	0.05803	0.59333	H475	0.80862	0.39541	0.2184
H476	0.55447	0.17137	0.79569	H476	0.82315	0.43463	0.09192
H477	0.53324	0.21165	0.81379	H477	0.85704	0.37663	0.74051
H478	0.5467	0.21837	0.39486	H478	0.85659	0.34051	0.81343
H479	0.5684	0.17715	0.37959	H479	0.85954	0.29915	0.85543
H480	0.57145	0.48418	0.726	H480	0.8773	0.2879	0.19534
H481	0.50733	0.45647	0.96066	H481	0.87638	0.3296	0.15191
H482	0.55051	0.41767	0.98732	H482	0.6973	0.03849	0.20685
H483	0.48693	0.4011	0.59401	H483	0.65051	0.96544	0.84629
H484	0.44121	0.43863	0.57101	H484	0.7088	0.92965	0.88279
H485	0.55007	0.33907	0.90744	H485	0.64735	0.90444	0.28631
H486	0.57288	0.29806	0.89217	H486	0.59315	0.94056	0.24359
H487	0.57116	0.30445	0.46983	H487	0.75577	0.89271	0.69934
H488	0.54944	0.34552	0.49146	H488	0.81547	0.8635	0.52912
H489	0.32704	0.49789	0.69122	H489	0.86272	0.82539	0.52229
H490	0.08667	0.38505	0.5	H490	0.67366	0.79359	0.59184
H491	0.56936	0.37815	0.90012	H491	0.62638	0.83163	0.59302
H492	0.54244	0.88832	0.4784	H492	0.78306	0.48623	0.42614
H493	0.60539	0.14111	0.38163	H493	0.78293	0.53201	0.42898
H494	0.11995	0.14491	0.76022	H494	0.8848	0.56828	0.67496
H495	0.57913	0.64289	0.91373	H495	0.84513	0.60645	0.7874
H496	0.05851	0.64778	0.59511	H496	0.79531	0.61831	0.16235

H497	-0.0976	0.0122	0.65491	H497	0.83233	0.58005	0.05319
C498	0.05329	0.00294	0.62946	H498	0.80336	0.64809	0.40823
C499	-0.64934	0.00403	0.68438	H499	0.80499	0.69582	0.20148
H500	0.33578	0.53391	0.71191	H500	0.78569	0.73614	0.30957
H501	0.79477	0.00749	0.61267	H501	0.75848	0.71601	0.94048
H502	1.0895	0.54401	0.67686	H502	0.77617	0.67552	0.83097
H503	0.58203	0.52918	0.73351	C503	0.47817	0.9476	0.52656
H504	0.77398	0.64485	0.4068	C504	0.50794	0.96875	0.48465
C505	0.27025	0.85922	0.35391	C505	0.57185	0.97503	0.53069
C506	0.27944	0.84595	0.4661	C506	0.65688	1.00659	0.5429
C507	0.29085	0.82256	0.45943	C507	0.59351	0.99849	0.5394
C508	0.2913	0.81101	0.33948	N508	0.55432	1.01664	0.54644
C509	0.27752	0.824	0.22785	H509	0.49602	0.93418	0.61808
C510	0.26773	0.84745	0.23482	H510	0.69945	0.99641	0.53818
H511	0.27932	0.85477	0.56081	H511	0.50432	1.01574	0.56136
H512	0.29967	0.81344	0.55116	H512	0.45662	0.51084	0.57192
H513	0.27643	0.81555	0.13243	N513	0.46945	0.04491	0.02611
H514	0.26077	0.85704	0.14428	N514	0.47297	0.98039	0.35511
H515	0.60913	0.98476	0.70563	N515	1.02465	0.04047	0.46531
H516	0.75824	0.98472	0.23681	N516	1.03076	0.97596	0.77582
H517	1.24877	0.99157	0.24857	N517	0.99666	0.54597	0.03602
H518	1.09679	0.9926	0.63256	N518	0.99114	0.47559	0.00803
H519	1.13074	0.88406	0.55023	N519	0.51453	0.4758	0.59476

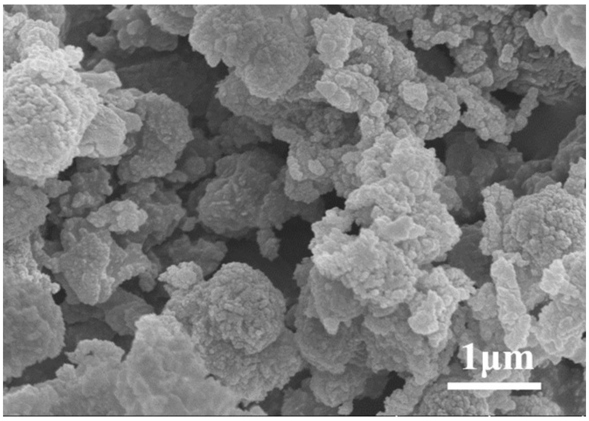

Fig. S2 SEM image of **DSPP-COF**.

6. Model Reaction

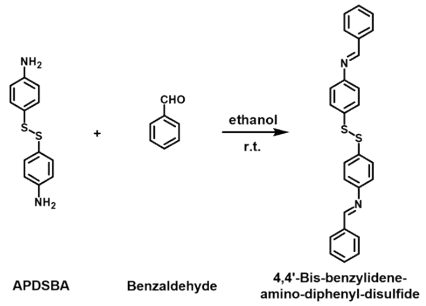

Synthesis. A mixture of APDSBA (425 mg, 1.0 mmol) and benzaldehyde (265 mg, 2.5 mmol) in ethanol (20 mL) was stirred for 4 h at room temperature. The resultant precipitates were filtered and washed with cold ethanol (three times) to afforded model compound as off-white solids. After recrystallized in dichloromethane/methanol (20 mL, 3:1, V/V), 4,4'-bis-benzylidene-amino-diphenyl-disulfide was obtained as the gray needle-like crystals in 65% yield. IR (KBr pellet cm^{-1}): 3062 (v), 2873 (v), 2850 (v), 1620 (s), 1570 (m), 1480 (m), 1450 (m), 1400 (v), 1360 (v), 1192 (m), 1168 (m), 1105 (v), 1073 (v), 1005 (v), 971 (v), 880 (m), 818 (s), 755 (s), 712 (v), 687 (s), 532 (m). ^{1}H NMR (400 MHz, chloroform-d) δ = 8.42 (s, 2H), 7.93-7.81 (m, 4H), 7.57-7.50 (m, 4H), 7.47 (d, J = 1.4, 4H), 7.45 (s, 2H), 7.15 (d, J = 8.4, 4H). HRMS (ESI) m/z $[M+H]^+$ calcd for $C_{26}H_{20}N_2S_2$ 425.1101, found 425.1114.

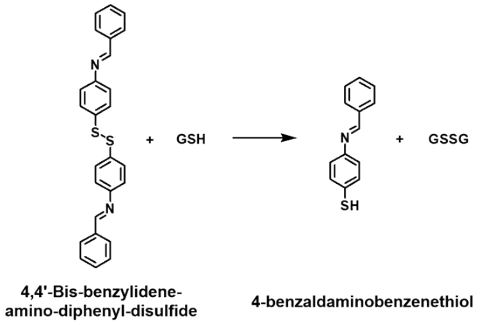

Degradation. A mixture of 10 µM 4,4'-bis-benzylidene-amino-diphenyl-disulfide and 10 mM GSH in PBS was stirred for 12 hours at 37 °C to afford degradation product in 93% yield. IR (KBr pellet cm^{-1}): 3394 (m), 3030 (v), 2947 (v), 2925 (v), 1625 (m), 1588 (s), 1397 (v), 1360 (v), 1313 (m), 1285 (m), 1248 (v), 1178 (m), 1122 (m), 1094 (m), 1073 (v), 1028 (v), 973 (v), 881 (v), 815 (m), 758 (w), 732 (v), 694 (m), 536 (v), 519 (v). ^{1}H NMR (400 MHz, $CDCl_3$) δ = 8.45 (s, 1H), 7.93 (m, 2H), 7.50 (m, 2H), 7.48 (m, 1H), 7.33-7.31 (m, 2H), 7.17-7.15 (2H), 3.49 (s,1H). HRMS (ESI) m/z $[M]^-$ calcd for $C_{13}H_{10}NS^-$ 212.0534, found 212.0533.

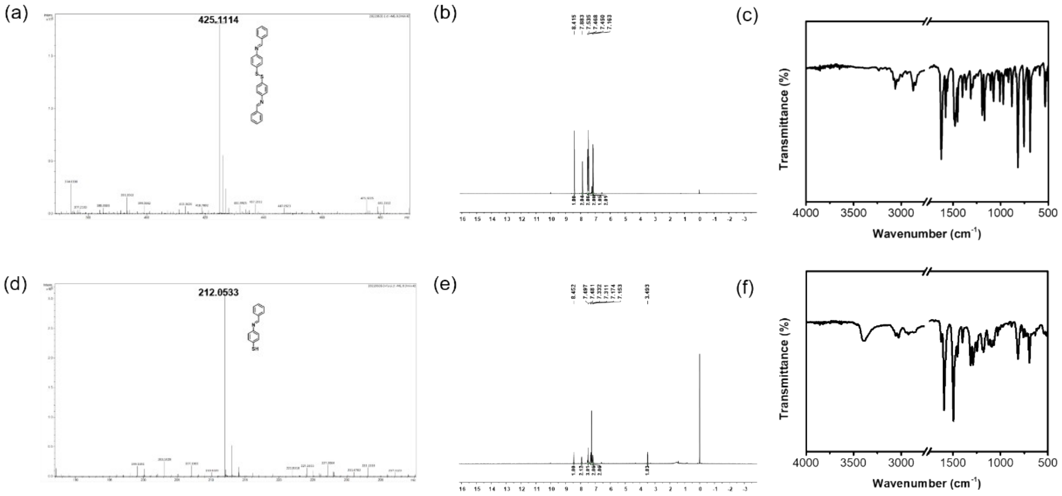

Fig. S3 (a) MS spectrum of 4,4'-bis-benzylidene-amino-diphenyl-disulfide. (b) ^{1}H NMR spectrum of 4,4'-bis-benzylidene-amino-diphenyl-disulfide. (c) IR spectrum of 4,4'-bis-benzylidene-amino-diphenyl-disulfide. (d) MS spectrum of 4-benzaldaminobenzenethiol. (e) ^{1}H NMR spectrum of 4-benzaldaminobenzenethiol. (f) IR spectrum of 4-benzaldaminobenzenethiol.

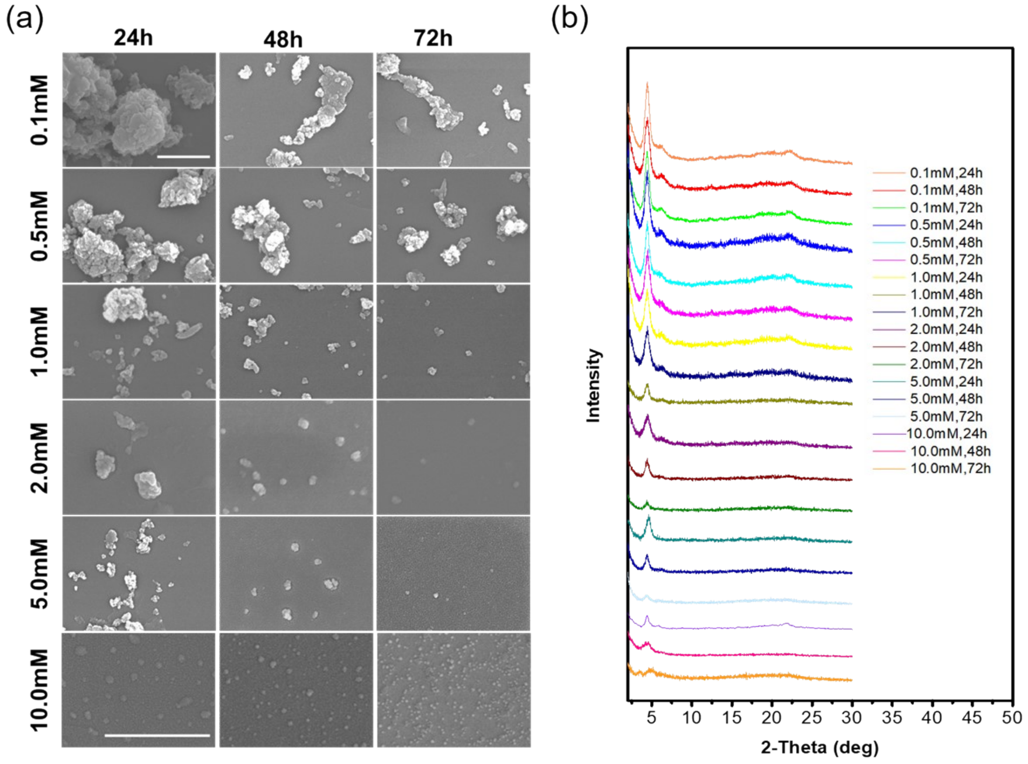

Fig. S4 (a) SEM images for **DSPP-COF** with GSH in PBS at different concentrations and times. Scale bar, 1 μm. The last three images share a scale bar with the 10 mM, 24 h image. (b) Corresponding PXRD patterns of **DSPP-COF** with GSH at different concentrations and times.

7. Synthesis of nano DSPP-COF

1 mg of **DCPP-COF** was dispersed into 10 mL of 10 mM glutathione PBS solution and stirred at 37 °C for 24 h. After filtration and removal of the residual GSH by washing, **nano DSPP-COF** was obtained.

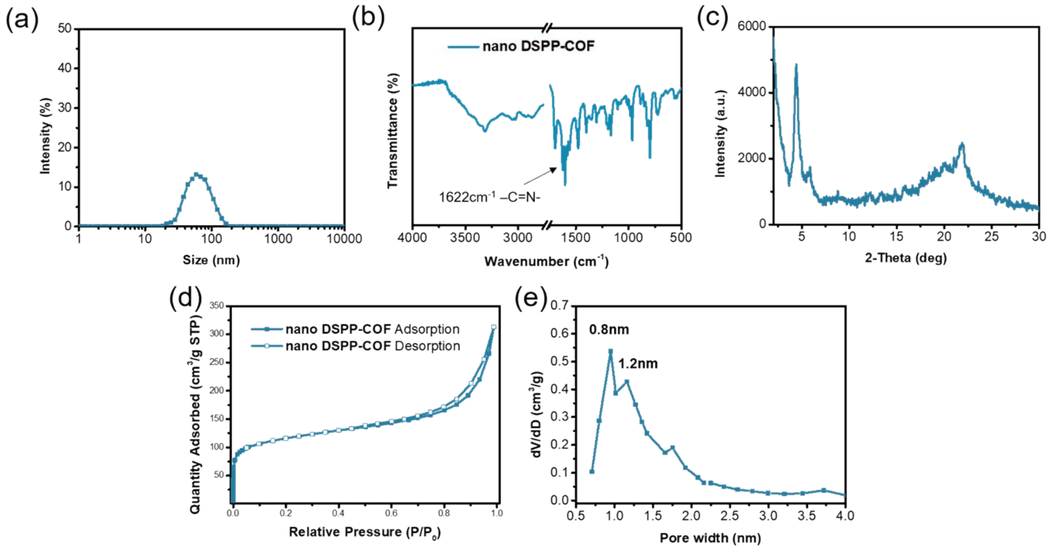

Fig. S5 (a) Intensity (%) vs size distribution of **nano DSPP-COF**. (b) FT-IR spectra of **nano DSPP-COF**. The peaks at 1622 cm^{-1} for -C=N. (c) PXRD pattern of **nano DSPP-COF**. (d) N_2 adsorption (solid circle) and desorption (hollow circle) isotherms of **nano DSPP-COF**. BET surface area of **nano DSPP-COF** = 424.3 m^2/g. (e) Pore size distributions of **nano DSPP-COF**. The pore size distribution curve based on nonlocal density functional theory (NLDFT) analysis, the pore width of **nano DSPP-COF** was centered at 0.8 and 1.2 nm.

8. Determination of sulfhydryl groups on nano DSPP-COF by Ellman's method

The Ellman reagent DTNB, i.e. 5,5'-dithiobis(2-nitrobenzoic acid), reacts with sulfhydryl groups to displace benzoic acid, i.e. TNB. DTNB has a characteristic absorption peak at 325 nm, while TNB shows a strong absorption peak at 412 nm under weakly basic conditions, and the sulfhydryl group concentration and absorbance values are in accordance with the Lambert-Beer law. The reaction of the sulfhydryl group with DTNB proceeded quantitatively, so the sulfhydryl group content in the sample could be determined by UV-Vis spectrophotometry.

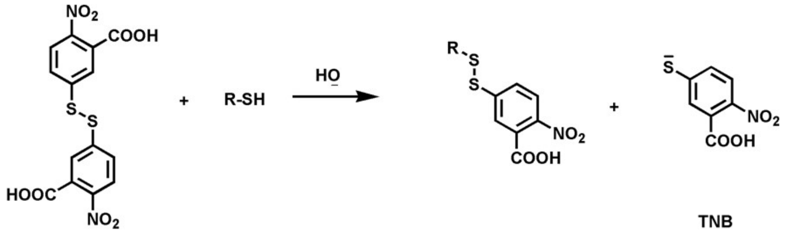

Ellman's reagent preparation: 2 mg of DTNB was dissolved in 2 mL of a buffer solution with pH 7.8.

Establishment of the standard curve: 4-Aminothiophenol was used as the sulfhydryl standard. 200 μL of 4-Aminothiophenol was mixed with 200 μL of Ellman's reagent and 1 mL of pH 7.8 buffer solution and incubated for 10 min at 37 °C. The absorbance at 412 nm was then measured using a multi-mode microplate detection system. Then the standard curve of concentration and absorbance was established.

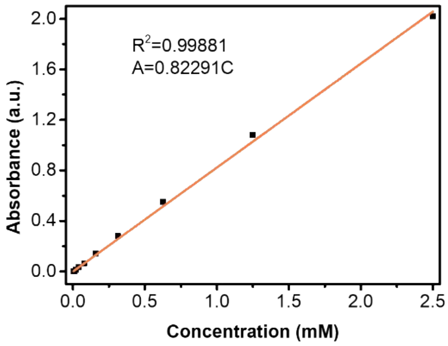

Fig. S6 Standard curve of TNB at 412 nm based on UV−vis spectra absorbance.

Preparation of the dispersion to be measured: 10 mg of **nano DSPP-COF** was dispersed in 1 mL of ethanol to obtain a dispersion of 10 mg/mL concentration.

200 μL of the above solution was mixed with 200 μL of Ellman's reagent and 1 mL of buffer solution and incubated at 37 °C for 10 min. The reaction solution was then centrifuged at 13300 rpm for 10 min and the supernatant was filtered through an aperture of 0.22 μm of microporous membrane filter. Then the absorbance at 412 nm was measured using a multi-mode microplate detection system.

9. Drug loading experiments

A mixture of **nano DSPP-COF** (5 mg) and 5-Fu (5 mg) in methanol (5 mL) was sonicated for 10 min. After being stirred (800 rpm) at 25 °C for 24 h, the resulting solids were separated by vacuum filtration and washed with water for 3 times. Finally, the obtained **5-Fu⊂nano DSPP-COF** was resuspended in water and stored at 4 °C until use.

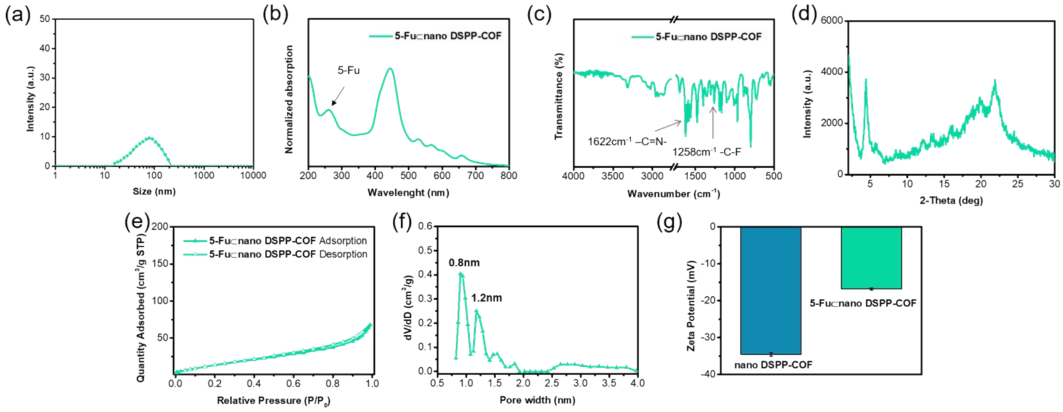

Fig. S7 (a) Intensity (%) vs size distribution of **5-Fu⊂nano DSPP-COF**. (b) Ultraviolet−visible (UV−vis) absorption spectrum of **5-Fu⊂nano DSPP-COF**. A characteristic absorption band of 5-Fu at 258 nm was observed. (c) FT-IR spectrum of **5-Fu⊂nano DSPP-COF**. The C-F stretching vibration in 5-Fu was found at 1258 cm^{-1}, and the -C=N stretching vibration was found at 1622 cm^{-1}. (d) PXRD pattern of **5-Fu⊂nano DSPP-COF**. (e) N_2 adsorption (solid circle) and desorption (hollow circle) isotherms of **5-Fu⊂nano DSPP-COF**. BET surface area of **5-Fu⊂nano DSPP-COF** is 61.6 m^2/g. The specific surface area significantly decreased after 5-Fu loading. (f) Pore size distributions of **5-Fu⊂nano DSPP-COF**. The pore size distribution curve based on nonlocal density functional theory (NLDFT) analysis, the pore width of **5-Fu⊂nano DSPP-COF** was centered at 0.8 and 1.2 nm respectively. Compared with **nano DSPP-COF**, the ratio of 1.2 nm pore to 0.8 nm pore

is significantly reduced. (g) Zeta potentials of **nano DSPP-COF**, and **5-Fu⊂nano DSPP-COF**. The negative zeta potential of **nano DSPP-COF** is due to the presence of a large number of sulfhydryl groups on its surface. The COF nanocarrier possessed a negative surface charge regardless of the 5-Fu loading. The increased zeta potential of **5-Fu⊂nano DSPP-COF** may be due to a slight increase in particle size after drug loading. [1]

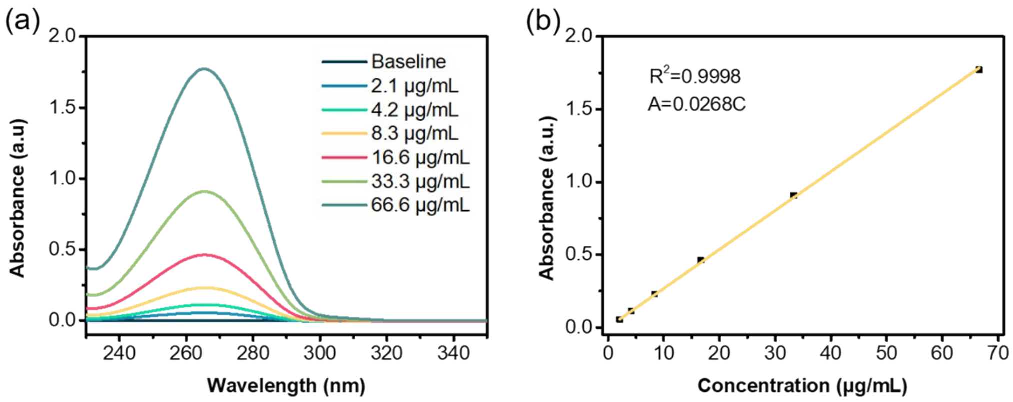

Fig. S8 (a) Ultraviolet−visible (UV−vis) absorption spectrum of different concentrations of 5-Fu in PBS buffer solution. (b) Standard curve of 5-Fu based on absorption spectra. To avoid the interference of buffer solutions, in the measurement of the Ultraviolet−visible absorption spectrum of 5-Fu, the detector conversion wavelength was set to 370 nm and the collection wavelength was set to 230-350 nm before the measurement to reduce the effect of the detector conversion on the spectrum. Next, the baseline was scanned with PBS, and then measured the Ultraviolet−visible absorption spectrum of PBS at 230-350 nm, when the absorption spectrum of PBS was a smooth straight line, and then performed the later measurements, to ensure the accuracy of the results.

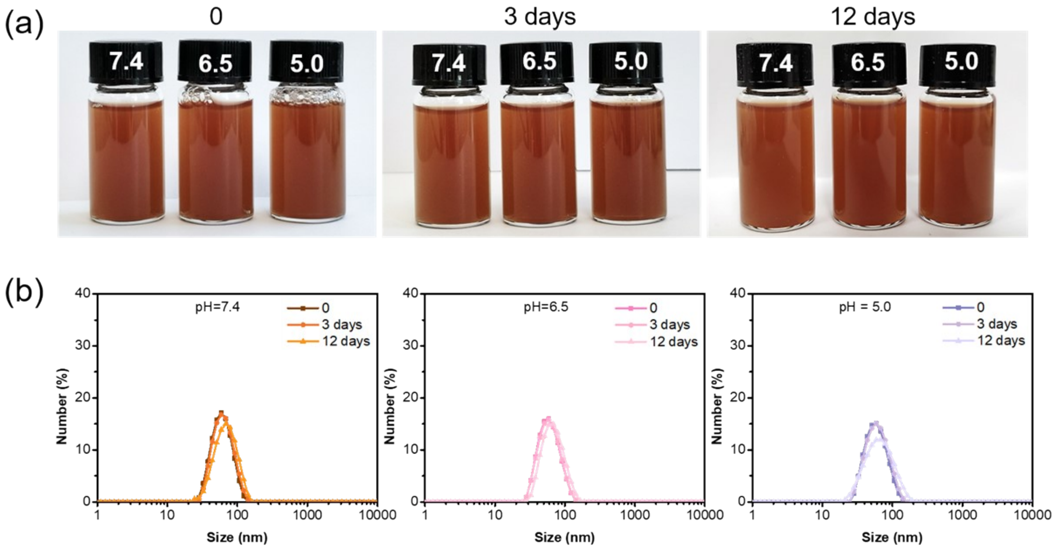

Fig. S9 (a) Digital photos of **5-Fu⊂nano DSPP-COF** after 0, 3 and 12 days in PBS at different pH values (pH = 7.4, 6.5, 5.0). (b) Particle size profiles of **5-Fu⊂nano DSPP-COF** measured by DLS in PBS at different pH values (pH = 7.4, 6.5, 5.0) after 0, 3, and 12 days.

10. Drug Release Experiments

5-Fu⊂nano DSPP-COF (8 mg) was suspended in 8 mL PBS with or without GSH. The system was maintained at a magnetic stirring speed of 100 rpm. Every once in a while, 3 mL of the above

dispersion liquids were taken out and centrifugated immediately, and refilled with the same volume of fresh PBS, the solids obtained by centrifugation were also put back together. The amount of released 5-Fu was quantified by absorption spectrum and the accumulated drug release (ADR) of 5-Fu was calculated according to the following formula:

$$\mathrm{ADR\ (wt\,\%)} = \left\{\left(8\mathrm{Cn} + 3\sum_{\mathrm{i}=n}^{\mathrm{n-1}}\mathrm{Ci}\right)/\mathrm{weight\ of\ loaded\ 5\text{-}Fu}\right\} \times 100\%$$

, where Cn is the concentration of 5-Fu in the supernatant at the time point of n.

To avoid the interference of the GSH solution, in the measurement of the Ultraviolet−visible absorption spectrum of 5-Fu, the detector conversion wavelength was set to 370 nm and the collection wavelength was set to 230-350 nm before the measurement to reduce the effect of detector conversion on the spectrum. Next, the baseline was scanned with GSH solution, and then measured the Ultraviolet−visible absorption spectrum of GSH solution at 230-350 nm, when the absorption spectrum of GSH solution was a smooth straight line, and then performed the later measurements, to ensure the accuracy of the results. Drug release experiments were performed three times under the same conditions.

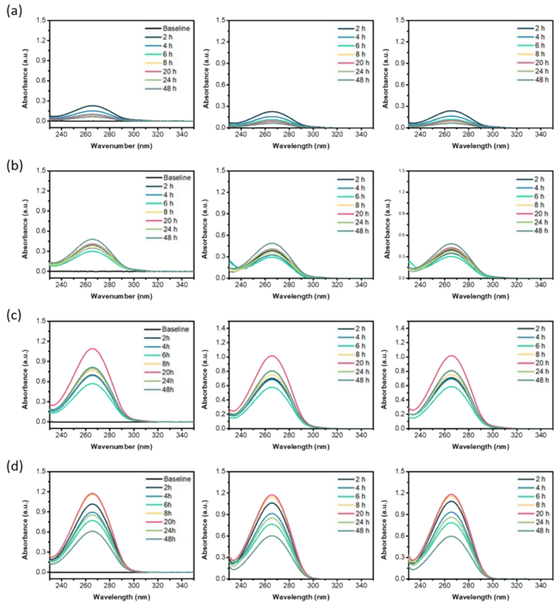

Fig. S10 Ultraviolet−visible (UV−vis) absorption spectrum of drug release at different time points in different media. (a) In PBS buffer solution. (b) In PBS buffer solution containing 1 mM GSH. (c) In PBS buffered solution containing 5 mM GSH. (d) In PBS buffer solution containing 10 mM GSH. Each group of experiments was repeated three times.

11. ESR-Trapping Test

The acetone dispersion of **nano DSPP-COF** or **5-Fu⊂nano DSPP-COF** (200 µL, 50 µg/mL) and 10 µL TEMP in a test tube was irradiated with a red LED (50 mW/cm^2) for 60 s. Then, the resulting system was characterized using a Bruker EMX plus model spectrometer operating at the X-band frequency (9.4 GHz) at room temperature.

12. Photodynamic Property

A mixture of **nano DSPP-COF** or **5-Fu⊂nano DSPP-COF** (2 mL, 50 µg/mL) and DPBF (200 µL, 1 mM) in DMF in a quartz dish was irradiated with a red LED (50 mW/cm^2) for 1 min. The absorbance of DPBF at 414 nm in the mixture was recorded at 10 s intervals. The $^1O_2$ generation

rate was determined from the reduced absorbance over time. To characterize the difference in the rate of $^1O_2$ introduced by different lasers, the ratios A/A_0 of absorbance A and the initial absorbance A_0 at 414 nm at different irradiation times were calculated and plotted as the ordinate for the irradiation time. The dispersion of **nano DSPP-COF** or **5-Fu⊂nano DSPP-COF** (2 mL, 50 µg/mL) was used as the reference for this UV−vis measurement.

13. Cell Uptake and Subcellular Localization

To study the cell uptake, intracellular distribution, and subcellular localization, **nano DSPP-COF** was labelled with the fluorescent dye Bodipy-CHO. Briefly, **nano DSPP-COF-Bodipy** was prepared as follows: a mixture of **nano DSPP-COF** (1 mg), Bodipy-CHO (1 mg), and acetic acid (20 µL, 6 M) in ethanol (2 mL) was stirred at 70 °C for 24 h in the dark. After fully washing with ethanol, the resulting solids were re-dispersed into DPBS (1 mL) to afford a stock solution of **nano DSPP-COF-Bodipy** (1 mg/mL). The structure of Bodipy-CHO and the schematic diagram of post-synthetic modifications are illustrated below:

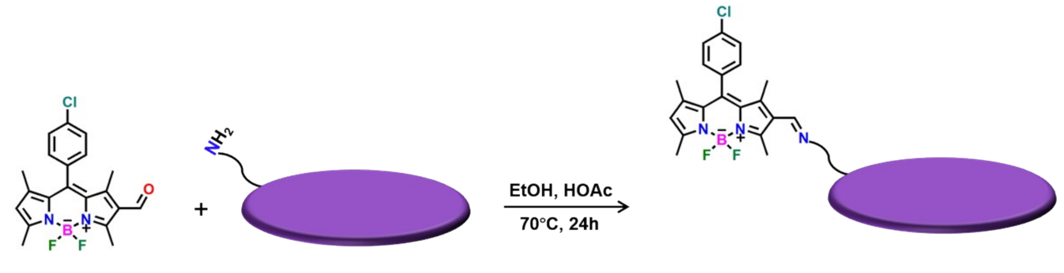

Fig. S11 The structure of Bodipy-CHO and schematic diagram of post-synthetic modifications.

For subcellular localization, cells were incubated with **nano DSPP-COF-Bodipy** (200 µL, 20 µg/mL) for 4 h in a CO_2 incubator, and carefully washed with DPBS twice. After additional 4 h incubation, cells were incubated with MitoTracker Deep Red FM (200 µL, 50 nM) for an additional 10 min, and washed with DPBS twice. Finally, the cells were washed with DPBS twice and the laser scanning confocal fluorescence images were captured. The images of **nano DSPP-COF-Bodipy** were excited by 488 nm light, and the emission wavelength range was collected at 520 ± 15 nm. The images of MitoTracker Deep Red FM were excited by 633 nm light, and the emission wavelength range was collected at 665 ± 15 nm. Controls were conducted as needed to make sure images were free of crosstalk. Colocalization was analyzed by ImageJ software.

14. Cellular Uptake Mechanism

MCF-7 Cells were subjected to different treatments before the incubation of **nano DSPP-COF-Bodipy** as follows: (i) DPBS, CO_2 incubator, 1 h; (ii) HBSS, air atmosphere, 4 °C, 1 h; (iii) sodium dichloroacetate (DCA), 15 mM, CO_2 incubator, 1 h; (iv) chlorpromazine (CPZ), 10 µg/mL, CO_2 incubator, 1 h; (v) methyl-β-cyclodextrin (MβCD), 10 mg/mL, CO_2 incubator, 1 h; (vi) amiloride (AMR), 37.5 µg/mL, CO_2 incubator, 1 h. After these different treatments, the cells were incubated with DPBS dispersion of **nano DSPP-COF-Bodipy** (200 µL, 20 µg/mL) in a CO_2 incubator for 4 h, and washed with DPBS twice. The laser scanning confocal fluorescence images were captured. The green images of **nano DSPP-COF-Bodipy** were excited by 488 nm light, and the emission wavelength range was collected at 520 ± 15 nm. The mean fluorescence intensity (MFI) was analyzed by ImageJ software.

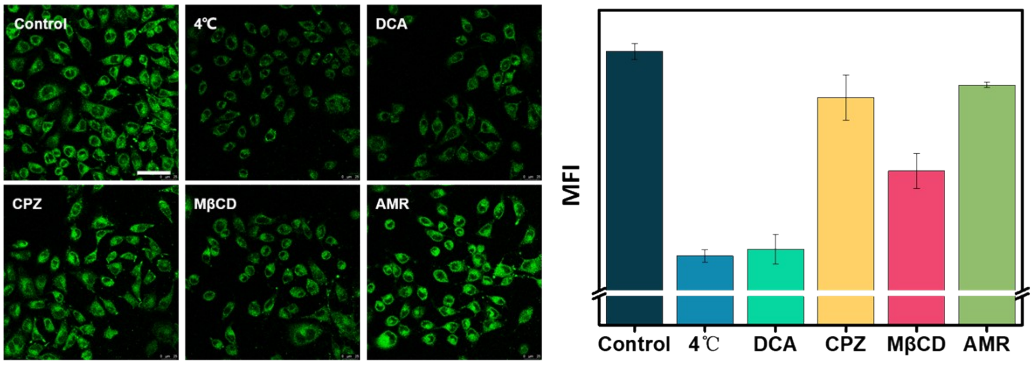

Fig. S12 Cellular uptake mechanism of **nano DSPP-COF-Bodipy** in MCF-7 cells. The cells were treated with **nano DSPP-COF-Bodipy** (20 µg/mL) for 4 h, at 37 °C, 4 °C (energy generation suppression), and 37 °C while pre-treating with DCA (15 mM, inhibiting aerobic glycolysis through inhibiting pyruvate dehydrogenase kinase), CPZ (10 µg/mL, clathrin-dependent endocytosis inhibitor), MβCD (10 mg/mL, caveolin-dependent endocytosis inhibitor), and AMR (37.5 µg/mL, micropinocytosis inhibitor) for 1 h. The cellular uptake was reflected by the MFI of green fluorescence. Data were presented as mean ± SD (n = 4). Scale bar, 50 µm.

15. Intracellular GSH Measurements

The level of intracellular GSH was measured using a GSH assay kit based on the 5,5'-dithiobis(2-nitrobenzoic acid) (DTNB) colorimetric method. Experimentally, cells were seeded and cultured in 60 mm culture dishes for 24 h and treated with **nano DSPP-COF** (2.0 mL, 10 µg/mL) for 4 h in a CO_2 incubator. After being rinsed with DPBS carefully, the cells were cultured for an additional 24, 48, or 72 h and taken for GSH measurements according to the manufacturer's guidelines of the assay kit. Colorimetric signals were measured by absorbance at 412 nm using a multi-mode microplate detection system. The GSH content was normalized to total protein amount of the cell lysates from a parallel plate and expressed as a percentage value relative to the control group value.

16. CCK-8 Cell Viability Assays

Standard CCK-8 assay was applied to evaluate the cell cytotoxicity of the nanodrugs. Experimentally, ~5000 cells were cultured in 96-well plates for 24 h and treated with **nano DSPP-COF** or **5-Fu⊂nano DSPP-COF** (100 µL, 0~20 µg/mL) for 4 h in a CO_2 incubator. Then, the cells were washed with DPBS carefully. For PDT, the cells were exposed to red LED (50 mW/cm^2, 8 min), and cultured for an additional 24 h. Subsequently, the CCK-8 solution (10 µL) was added to each well and the plate was incubated in a CO_2 incubator for about 2 h. The absorbance at 450 nm was determined using a multi-mode microplate detection system.

For the selectivity of **5-Fu⊂nano DSPP-COF** on normal and cancer cells, cell viabilities of MCF-7 and MCF-10A cells were treated with **5-Fu⊂nano DSPP-COF** for 4 h and cultured for an additional 72 h. Subsequently, the CCK-8 solution (10 µL) was added to each well and the plate was incubated in a CO_2 incubator for about 2 h. The absorbance at 450 nm was determined using a multi-mode microplate detection system.

For the toxicity of 5-Fu, we incubated the MCF-7 cells with the same concentration of 5-Fu as in **5-Fu⊂nano DSPP-COF** for 4 h and continued incubation for 24 h after the drug was removed. Subsequently, the CCK-8 solution (10 µL) was added to each well and the plate was incubated in a CO_2 incubator for about 2 h. The absorbance at 450 nm was determined using a multi-mode microplate detection system.

In the CCK-8 cell viability assay, the cells without treatment were used as the control. The wells without cells were used as blanks. The cell viability was expressed as a percentage value relative to the control group value.

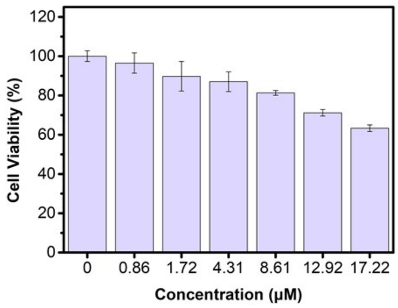

Fig. S13 Cytotoxicity of MCF-7 with the same concentration of 5-Fu as in **5-Fu⊂nano DSPP-COF**.

17. Calcein-AM/PI Double Staining

Cells were seeded into 60 mm culture dishes and incubated overnight in a CO_2 incubator. After removal of the culture medium, the cells were incubated with DPBS dispersion of **nano DSPP-COF** or **5-Fu⊂nano DSPP-COF** (2 mL, 20 μg/mL) for 4 h in a CO_2 incubator. For PDT, the cells were exposed to red LED (50 mW/cm^2, 8 min). For the Fer-1 addition group, MCF-7 cells were first pretreated with Fer-1 at a concentration of 20 μM for 1 h, followed by the same treatment as above. After additional 24 h incubation, cells were collected by centrifugation after digestion with trypsin solution, washed with DPBS twice carefully, and incubated with calcein-AM (500 μL, 2 μM) and PI (500 μL, 4 μM) for 15 min. Finally, the cells were washed with DPBS twice carefully, and imaged with a laser scanning confocal microscope. The green images of living cells were excited by 488 nm light, and the emission wavelength range was collected at 520 ± 20 nm. The red images of dead cells were excited by 514 nm light, and the emission wavelength range was collected at 640 ± 20 nm.

18. Intracellular Total ROS Measurements

Levels of intracellular ROS were measured by the cell-permeable dye DCFH-DA. Experimentally, cells were treated with **nano DSPP-COF** or **5-Fu⊂nano DSPP-COF** (500 μL, 20 μg/mL) for 4 h in a CO_2 incubator. For the Fer-1 addition group, MCF-7 cells were first pretreated with Fer-1 at a concentration of 20 μM for 1 h, followed by the same treatment as above. And then the cells were washed with DPBS carefully. Afterward, the cells were loaded with DCFH-DA (200 μL, 100 nM) for 15 min in a CO_2 incubator and washed with DPBS twice. For PDT, the cells were exposed to red LED (50 mW/cm^2, 8 min). Finally, the laser scanning confocal fluorescence images were captured. The green images were excited by a 488 nm light, and the emission wavelength range was collected at 525 ± 20 nm. Cells without any nanodrug treatment were used as a control group.

19. Cell Death Rescue Experiments

For the experiment on the effect of GSH-OET on cell viability, cells were cultured in 96-well plates for 12 h. The cells were pretreated with GSH-OEt (100 μL, 1 mM) for 2 h, and then treated with **nano DSPP-COF** (100 μL, 0-20 μg/mL) or **5-Fu⊂nano DSPP-COF** (100 μL, 0-20 μg/mL) for 4 h in a CO_2 incubator. For PDT, the cells were exposed to red LED (50 mW/cm^2, 8 min). After additional 24 h incubation, the CCK-8 solution (10 μL) was added to each well and the plate was incubated in a CO_2 incubator for 2 h. The absorbance at 450 nm was determined using a multi-mode

microplate detection system.

For cell death rescue experiments, cells were cultured in 96-well plates for 12 h. The designated modulating compound including ferrostatin-1 (100 μL, 0~20 μM), liproxstatin-1 (100 μL, 0~5 μM), necrostatin-1 (100 μL, 0~1000 nM), 3-MA (100 μL, 0~100 μM) were added to each well, pretreated the cells for two hours. And then treated with **nano DSPP-COF** (100 μL, 20 μg/mL) or **5-Fu⊂nano DSPP-COF** (100 μL, 10 μg/mL) for 4 h in a CO_2 incubator. For PDT, the cells were exposed to red LED (50 mW/cm^2, 8 min). After additional 24 h incubation, the CCK-8 solution (10 μL) was added to each well and the plate was incubated in a CO_2 incubator for 2 h. The absorbance at 450 nm was determined using a multi-mode microplate detection system. The wells without cells were used as blanks.

The cells without treatment were used as the control. The cell viability was expressed as a percentage value relative to the control group value.

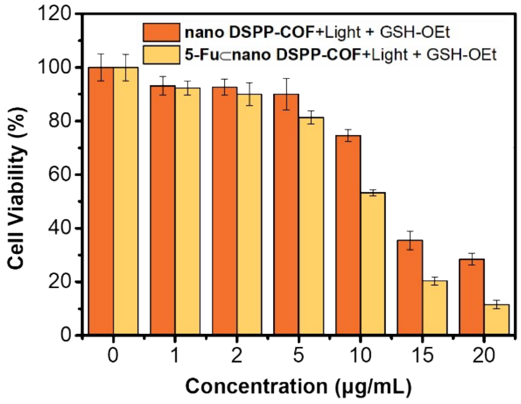

Fig. S14 Phototoxicity of **nano DSPP-COF** and **5-Fu⊂nano DSPP-COF** on MCF-7 cells in the presence of GSH-OE (100 μL, 1.0 mM).

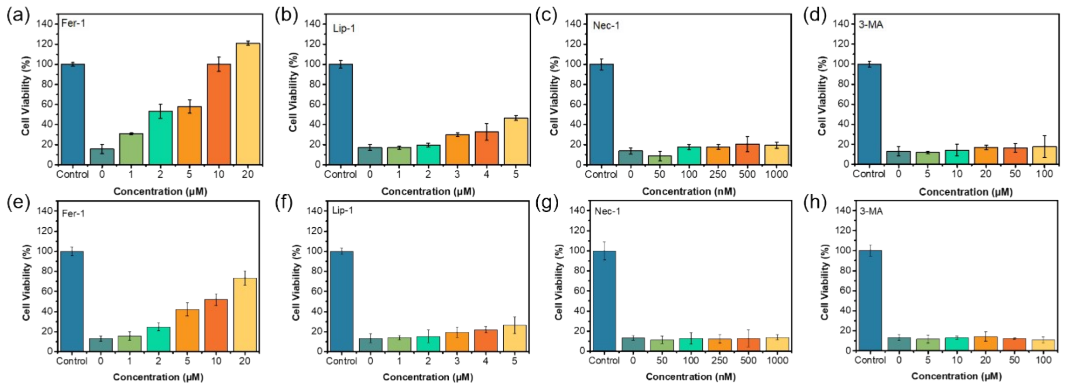

Fig. S15 (a)-(d) Effects of different inhibitors on **nano DSPP-COF** + Light induced cell death in MCF-7 cells. (e)-(f) Effects of different inhibitors on **5-Fu⊂nano DSPP-COF** induced cell death in MCF-7 cells. Ferrostatin-1 (100 μL, 0~20 μM), liproxstatin-1 (100 μL, 0~5 μM), necrostatin-1 (100 μL, 0~1000 nM), 3-MA (100 μL, 0~100 μM).

20. Intracellular GPX4 Activity Measurements

Cells were seeded and cultured in 60 mm culture dishes for 24 h and treated with **nano DSPP-COF** or **5-Fu⊂nano DSPP-COF** (2.0 mL,20 μg/mL) for 4 h in a CO_2 incubator. After carefully rinsed with DPBS. For PDT, the cells were exposed to a red LED (50 mW/cm^2, 8 min). After that, the cells were cultured for an additional 24 h, and then quickly frozen in liquid nitrogen. Subsequently, the

frozen cells were homogenized in lysis buffer on ice and the supernatant was used for GPX4 activity measurements using a glutathione peroxidase assay kit. Colorimetric signals were measured by absorbance at 340 nm using a multi-mode microplate detection system. The GPX4 activity was normalized to the total protein amount of the cell lysates from a parallel plate and expressed as a percentage value relative to the control group value. Similarly, the expression of GPX4 in MCF-7 cells upon formulation treatment was also analyzed by western blotting. The cell lysates containing identical protein (40 μg) were subjected to standard electrophoresis, followed by antibody incubation at 4°C. The dilution ratio for the first antibody was 1:2000 (actin-specific antibody) and 1:2500 (GPX4-specific antibody). Regarding the secondary antibody, the dilution ratio was 1:5000 for both GPX4 and actin. The protein bands were developed via the ECL™ western blotting detection reagents.

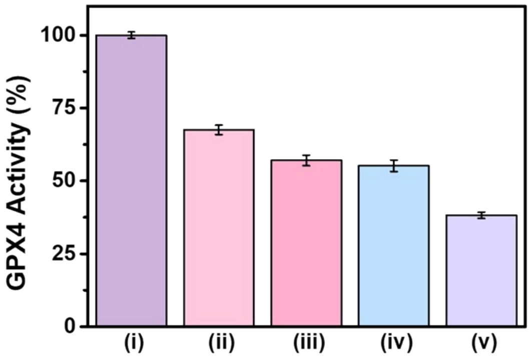

Fig. S16 GPX4 activity of MCF-7 cells that were treated with **nano DSPP-COF** and **5-Fu⊂nano DSPP-COF** with/without light. (i-v) in the figure represent (i) control, (ii) **nano DSPP-COF**, (iii) **5-Fu⊂nano DSPP-COF**, (iv) **nano DSPP-COF** + light, (v) **5-Fu⊂nano DSPP-COF** + light, respectively.

21. Intracellular Lipid Peroxidation Assays

Cells were treated with **nano DSPP-COF** or **5-Fu⊂nano DSPP-COF** (500 μL, 20 μg/mL) for 4 h in a CO_2 incubator. For PDT, the cells were exposed to red LED (50 mW/cm^2, 8 min). For the Fer-1 addition group, MCF-7 cells were first pretreated with Fer-1 at a concentration of 20 μM for 1 h, followed by the same treatment as above. And then the cells were washed with DPBS carefully and cultured for an additional 4 h. After that, the cells were incubated with C_{11}-BODIPY (200 μL, 2.0 μM) for 30 min in a CO_2 incubator and washed with DPBS twice. Finally, the laser scanning confocal fluorescence images were captured. The green images of the oxidized C_{11}-BODIPY dye were excited by a 488 nm light, and the emission wavelength range was collected at 510 ± 20 nm. The red images of the reduced C_{11}-BODIPY dye were excited by a 561 nm light, and the emission wavelength range was collected at 591 ± 20 nm. Cells without any nanodrug treatment were used as a control group.

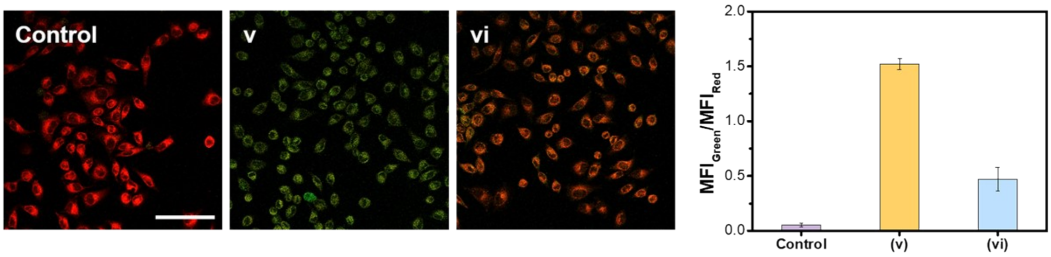

Fig. S17 LCSM images of MCF-7 cells under different treatment conditions for the detection of

intracellular lipid peroxidation using C_{11}-Bodipy. (v) **5-Fu⊂nano DSPP-COF** + light, (vi) **5-Fu⊂nano DSPP-COF** + light + Fer-1. Scale bar, 100 μm.

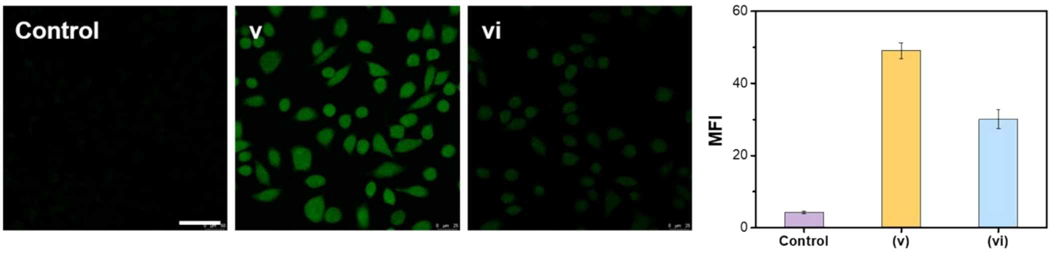

Fig. S18 Detection of intracellular $^1O_2$ using DCFH-DA. (v) **5-Fu⊂nano DSPP-COF** + light, (vi) **5-Fu⊂nano DSPP-COF** + light + Fer-1. Scale bar, 100 μm.

22. Mitochondrial Membrane Potential Measurements

Mitochondrial membrane potential was measured by a fluorescent lipophilic carbocyanine dye JC-1. Experimentally, cells were treated with **nano DSPP-COF** or **5-Fu⊂nano DSPP-COF** (500 μL, 20 μg/mL) for 4 h in a CO_2 incubator. And then the cells were washed with DPBS carefully. For the Fer-1 addition group, MCF-7 cells were first pretreated with Fer-1 at a concentration of 20 μM for 1 h, followed by the same treatment as above. For PDT, the cells were exposed to red LED (50 mW/cm^2, 8 min). After additional 4 h incubation, the cells were incubated with JC-1 (200 μL, 15 μM) for 10 min in a CO_2 incubator and washed with DPBS twice. Finally, the laser scanning confocal fluorescence images were captured. The green images of monomer were excited by 488 nm light, and the emission wavelength range was collected at 530 ± 15 nm. The red images of J-aggregate were excited by 561 nm light, and the emission wavelength range was collected at 590 ± 17 nm. Cells without any nanodrug treatment were used as a control group.

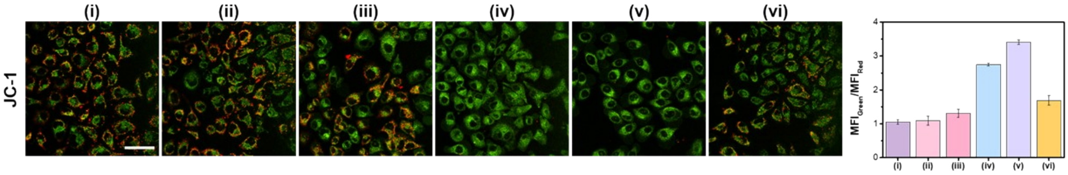

Fig. S19 Laser scanning confocal fluorescence microscopy images of JC-1 staining for determining mitochondrial membrane potential. (i-v) in the figure represent (i) control, (ii) **nano DSPP-COF**, (iii) **5-Fu⊂nano DSPP-COF**, (iv) **nano DSPP-COF** + light, (v) **5-Fu⊂nano DSPP-COF** + light, (vi) **5-Fu⊂nano DSPP-COF** + light + Fer-1, respectively. Scale bar, 50 μm.

23. Lysosomal Membrane Permeabilization Detections

Lysosomal membrane permeabilization was measured by a lysosomotropic metachromatic fluorochrome AO. Experimentally, cells were treated with various **nano DSPP-COF** or **5-Fu⊂nano DSPP-COF** (500 μL, 20 μg/mL) for 4 h in a CO_2 incubator. And then the cells were washed with DPBS carefully. For the Fer-1 addition group, MCF-7 cells were first pretreated with Fer-1 at a concentration of 20 μM for 1 h, followed by the same treatment as above. For PDT, the cells were exposed to red LED (50 mW/cm^2, 8 min). After additional 4 h incubation, the cells were incubated with AO (200 μL, 15 μM) for 10 min in a CO_2 incubator and washed with DPBS twice. Finally, the laser scanning confocal fluorescence images were captured. The green images of deprotonated AO were excited by 488 nm light, and the emission wavelength range was collected at 530 ± 20 nm. The red images of protonated AO were excited by 488 nm light, and the emission wavelength range was collected at 640 ± 20 nm. Cells without any nanodrug treatment were used as a control group.

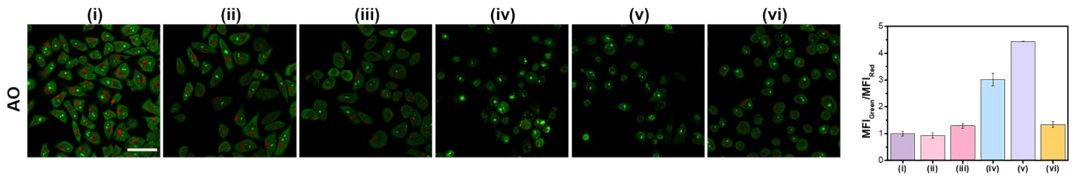

Fig. S20 Laser scanning confocal fluorescence microscopy images of AO staining for determining lysosomal membrane permeabilization. Scale bar, 50 μm. (i-v) in the figure represent (i) control, (ii) **nano DSPP-COF**, (iii) **5-Fu⊂nano DSPP-COF**, (iv) **nano DSPP-COF** + light, (v) **5-Fu⊂nano DSPP-COF** + light, (vi) **5-Fu⊂nano DSPP-COF** + light + Fer-1, respectively. Scale bar, 50 μm.

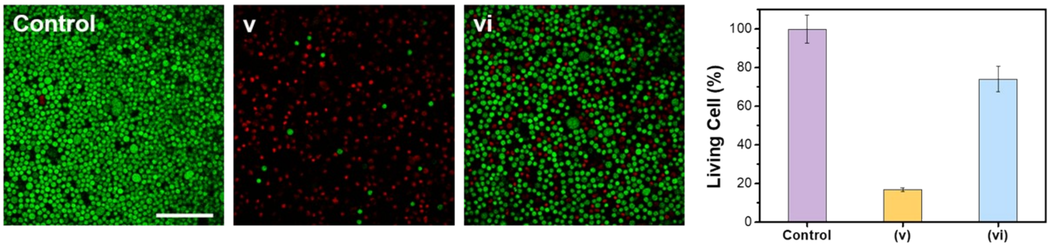

Fig. S21 Calcein-AM/PI double staining. (v) **5-Fu⊂nano DSPP-COF** + light, (vi) **5-Fu⊂nano DSPP-COF** + light + Fer-1. Scale bar, 200 μm.

24. Hemolysis Analysis

First, fresh nude mouse blood samples (2 mL) were added to PBS solution (4 mL), and red blood cells (RBC) were separated by centrifugation at 3000 rpm for 10 minutes. After washing 5 times with PBS, dilute purified red blood cells to 20 mL with PBS. For hemolysis assay, 0.2 mL of diluted RBCs suspension was added to 1.0 mL of PBS as a negative control and 1.0 mL of deionized water as a positive control. And 1.0 mL **5-Fu⊂nano DSPP-COF** suspension at a concentration range of 1 to 200 μg/mL. All mixtures were then allowed to stand at 37 °C for 5 h and then centrifuged at 13300 rpm for 10 minutes. Due to the small size of **5-Fu⊂nano DSPP-COF**, it was difficult to separate **5-Fu⊂nano DSPP-COF** completely even by centrifugation at 13300 rmp for 10 minutes. Therefore, we chose the supernatant of the corresponding concentration as a control. The absorbance of 541 nm supernatant was measured by synergy SpectraMax i3x multi-mode microplate reader. The hemolytic percentage of red blood cells was calculated by the following formula: Hemolysis Rate = [(Dt – Dcc)/(Dpc – Dnc)] ×100%.

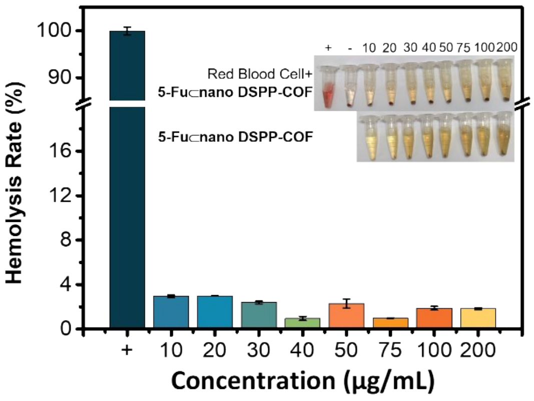

Fig. S22 Hemolytic assay using red blood cells incubated with control solvents and different concentrations of **5-Fu⊂nano DSPP-COF**.

25. In vivo biodistribution

To study in vivo biodistribution, **nano DSPP-COF** was labelled with the fluorescent dye Cy5-NH_2. Briefly, **nano DSPP-COF-Cy5** was prepared as follows: a mixture of **nano DSPP-COF** (1.0 mg), Cy5-NH_2 (1.0 mg), and acetic acid (20 µL, 6.0 M) in ethanol (2.0 mL) was stirred at 70 °C for 24 h in the dark. After fully washing with ethanol, the resulting solids were re-dispersed into DPBS (1.0 mL) to afford a stock solution of **nano DSPP-COF-Cy5** (1.0 mg/mL). **5-Fu⊂nano DSPP-COF-Cy5** was prepared by the same method as that used to prepare **5-Fu⊂nano DSPP-COF**, and the resulting solids were re-dispersed into DPBS (1.0 mL) to afford a stock solution of **5-Fu⊂nano DSPP-COF-Cy5** (1.0 mg/mL).

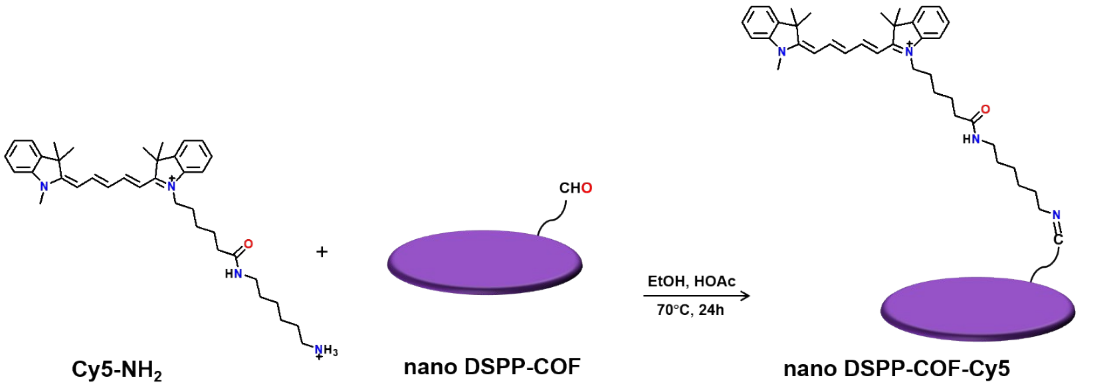

Fig. S23 The structure of Cy5-NH_2 and schematic diagram of post-synthetic modifications.

To evaluate the biodistribution of **5-Fu⊂nano DSPP-COF-Cy5** (50 µL,1mg /mL) was intratumorally injected. Then, nude mice with MCF-7 tumors were anesthetized and imaged at different times (4 h, 8 h, 12 h, 24 h, and 0-12 days) using an *in vivo* imaging system with an excitation wavelength of 640 nm and an emission wavelength of 680 nm. Then, the mice were killed and organs and tumors were separated for *ex vivo* imaging to determine the biodistribution pattern and retention of **5-Fu⊂nano DSPP-COF-Cy5** in the tumors. It is important to note that nude mice have to be fed with non-fluorescent chow for 24 hours before imaging.

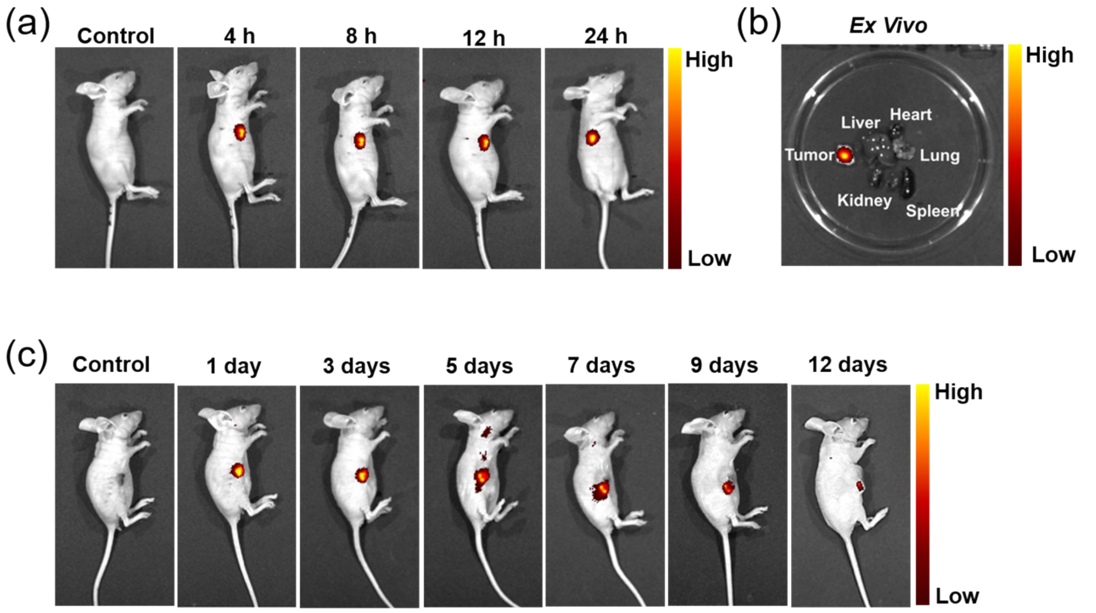

Fig. S24 *In vivo* and *ex vivo* imaging experiments. (a) Whole-body fluorescence images of MCF-7

tumor-bearing mice intratumorally injected with **5-Fu⊂nano DSPP-COF-Cy5** at different time points. (b) *Ex vivo* fluorescence imaging of each organ at 24 h. (c) Long-term whole-body fluorescence imaging assay in nude mice. 'Low' represents a fluorescence intensity of $9*10^6$, and 'High' represents a fluorescence intensity of $2.2*10^7$.

26. In Vivo Antitumor Therapy

MCF-7 cancer cells (10^6 cells) suspended in DPBS (100 μL) were subcutaneously injected into the flanks of each nude mice to establish the MCF-7 xenograft model. The length (L) and width (W) of the tumor were determined by digital calipers. The tumor volume (V) was calculated by the formula $V = 1/2\times L\times W^2$. When the tumor size reached ~100 mm^3, the nude mice bearing MCF-7 tumors (n = 30) were randomly distributed into six groups, i.e., (i) control, (ii) **nano DSPP-COF**, (iii) **5-Fu⊂nano DSPP-COF**, (iv)**nano DSPP-COF +** light, (v) **5-Fu⊂nano DSPP-COF +** light, and (vi) **5-Fu⊂nano DSPP-COF +** light + Fer-1 groups. After intratumoral injection PBS (100 μL), **nano DSPP-COF** or **5-Fu⊂nano DSPP-COF** (50 μL, 1 mg mL^{-1}), the nude mice were fed for 4 h, and for the treatment group, light treatment (660 nm laser, 100 mW cm^{-2}, 8 min) was performed on the tumor site. For **5-Fu⊂nano DSPP-COF +** light + Fer-1 groups, after injection **5-Fu⊂nano DSPP-COF**, Fer-1 (5.0 mg/kg) was injected. The mice continued to be fed for 12 days. The tumor volume and nude mouse body weight were recorded every other day during the experimental period.

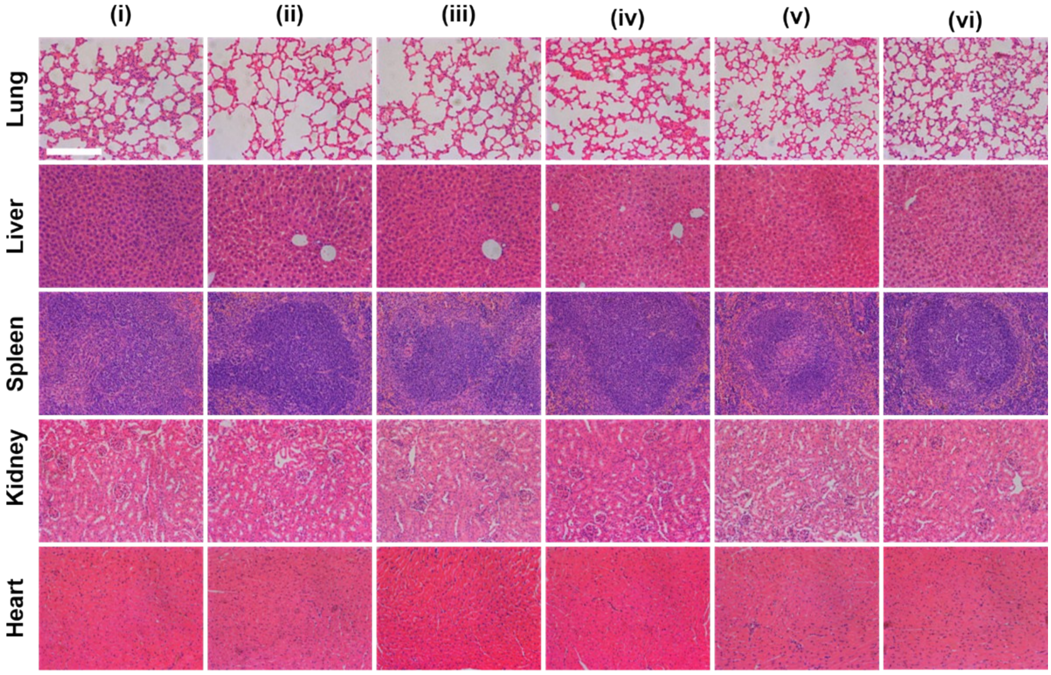

Fig. S25 H&E stained tissue sections from the lung, liver, spleen, kidney, and heart, of the nude mice at the end of the treatment. Scale bar, 200 μm. (i-v) in the figure represent (i) control, (ii) **nano DSPP-COF**, (iii) **5-Fu⊂nano DSPP-COF**, (iv) **nano DSPP-COF** + light, (v) **5-Fu⊂nano DSPP-COF** + light, (vi) **5-Fu⊂nano DSPP-COF** + light + Fer-1, respectively.

(a)

Group	Degree of damage
(i)	+
(ii)	+
(iii)	++
(iv)	++
(v)	+++
(vi)	++

(b)

Group	Proliferation Ability
(i)	+++
(ii)	+++
(iii)	++
(iv)	++
(v)	+
(vi)	++

(c)

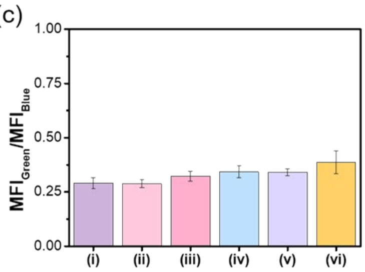

Fig. S26 (a) Evaluation of the degree of tumor tissue damage derived from H&E. In which, +++ indicates severe damage, ++ indicates slight damage, and + indicates almost no damage. (b) Evaluation of tumor proliferative ability derived from Ki67. Where, +++ indicates strong proliferative ability, ++ indicates medium proliferative ability, and + indicates weak proliferative ability. (c) MFI_{Green}/MFI_{Blue} of tunel.

27. Supplemental References

[1] (a) T. G. a. J. B. Talbot, *J. Electrochem. Soc.,* 2006, **153**, G622-G625. (b) N. Yeole, D. Hundiwale, *Colloids and Surfaces A: Physicochem. Eng. Aspects,* 2011, **392**, 329-334.
